# Simultaneous quantification of all B vitamins and selected biosynthetic precursors in seawater and bacteria by means of different mass spectrometric approaches

**DOI:** 10.1007/s00216-022-04317-8

**Published:** 2022-10-05

**Authors:** Stefan Bruns, Gerrit Wienhausen, Barbara Scholz-Böttcher, Heinz Wilkes

**Affiliations:** Institute for Chemistry and Biology of the Marine Environment (ICBM), Carl von Ossietzky University of Oldenburg, 26129 Oldenburg, Germany

**Keywords:** North Sea, *Phaeobacter inhibens*, Orbitrap Fusion, TSQ Quantum, B vitamin

## Abstract

B vitamins have high microbiological relevance in the marine environment, but their very low concentrations and the chemical heterogeneity of the individual vitamins make their analysis challenging. Mass spectrometric analysis of B vitamins in environmental samples at trace levels has mainly been performed using triple quadrupole mass spectrometers operated in targeted analysis mode. The development of such a method can be laborious and error prone. Additionally, high-resolution mass spectrometers can be used to measure a sample in full scan mode and subsequently search the total ion current chromatogram for extracted ion chromatograms of targeted vitamins. Three different analytical approaches for trace analysis of all B vitamins and some of their biosynthetic precursors were optimized and compared on two different mass spectrometers. A triple quadrupole mass spectrometer in selected reaction monitoring mode, and a high-resolution orbitrap mass spectrometer in parallel reaction monitoring, as well as in full scan mode were employed. Detection limits down to 10 ng/L were achieved with all three techniques. The methods were applied to a marine water sample from the North Sea and to the cell extract of a bacterial culture of *Phaeobacter inhibens*. Most vitamins and precursors were found in the bacterial cell extract and the seawater sample with all three measuring methods. The results of this study emphasize that, in addition to tandem mass spectrometry, high-resolution full scan mass spectrometry is a promising technique for the simultaneous detection of structurally diverse B vitamins in complex natural samples. This enables highly sensitive measurements without loss of detailed mass spectrometric information, which is inevitable when using a triple quadrupole system in MS/MS mode.

**Graphical abstract:**

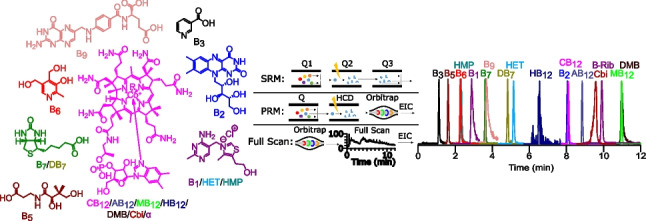

**Supplementary Information:**

The online version contains supplementary material available at 10.1007/s00216-022-04317-8.

## Introduction

All B vitamins are characterized by the fact that they are coenzymes or serve as precursors to coenzymes. Their grouping is based on a biochemical function and the individual B vitamins are structurally highly diverse. They span a broad range of polarities and cover molecular masses from 123 to 1579 daltons. This heterogeneity makes them difficult to analyze simultaneously, with the result that natural samples, especially when trace levels are to be expected, are often analyzed for one particular vitamin only or a limited number of vitamins [1, 2]. The main application area for B vitamin analysis is in food chemistry. Here, in particular regulated formulations like infant formula [3] or beverages fortified with vitamins [4] are investigated. In medical approaches, vitamin levels are of interest, e.g., in blood plasma analysis to evaluate possible vitamin deficiencies or the risk of colorectal cancer [1, 5, 6]. In contrast to vitamin-fortified formulations, values of plasma concentrations are much lower and are around 400 ng/L for B_12_ [5, 6] and 1400–6500 ng/L for other B vitamins (B_2_, B_6_, B_9_) [1, 6]. The analysis of B vitamins in seawater is of increasing interest as numerous microbial vitamin cross-feeding interactions occur and their availability even affects marine microbial communities [7, 8]. Thus, the content of B vitamins in the sea is closely linked to the occurrence of microbial producers and consumers [9, 10]. Only few studies on B vitamin quantification in seawater have been conducted [11–14]. Concentrations ranged between 0.1 and 16.2 ng/L (B_12_) [11, 12] or 0.1 and 48.2 ng/L (B_1_, B_2_, B_5_, B_6_, B_7_, HMP, DB_7_) [11, 12, 14] and have been almost one order of magnitude below B vitamin concentrations in plasma.

Since long, B vitamins have been analyzed by high-performance liquid chromatography (HPLC), however, the preferred detection methods have shifted over time from UV/Vis and fluorescence detectors to tandem mass spectrometry (MS/MS), which is currently the most common technique used to quantify B vitamins in natural samples [12, 15, 16]. In general, HPLC/MS/MS is widely used in quantitative trace analysis of polar compounds. Triple quadrupole mass spectrometers (QqQMS) in selected reaction monitoring (SRM) mode enable to detect and quantify trace amounts of targeted analytes in a wide variety of samples [12, 17, 18]. This approach has high selectivity using analyte-specific signals, while eliminating interfering matrix background. For the analysis of B vitamins in seawater samples, detection with QqQMS is the state of the art [11, 12]. A well-designed and -evaluated SRM method is required to ensure high data quality. In a step-by-step approach, the retention time windows of all respective analytes have to be individually determined. Considering the quite different stabilities of the B vitamins, any decomposition effects but also unintended adduct species formation have to be monitored and fragment ion intensities have to be individually and systematically optimized by collision energy variations. Any deviation from this systematic approach may result in individual analytes not being detected. In SRM mode, only pre-selected mass transitions are observed. Masses of other, untargeted compounds get lost. Incorrect fragment ion masses or retention time windows lead to false negative results, even if the analyte is present in the sample, or might lead conversely to a signal appearance even though the analyte is not present (false positive) [19].

Until recently, SRM was the only way to measure low-level analytes in complex and matrix-rich samples. Any full scan MS technique using single quadrupole, ion trap and time-of-flight (TOF) mass spectrometers could not compete with respect to selectivity and sensitivity. The advent of high-resolution mass spectrometers (HRMS), such as high-resolution time of flight (HRTOF) and Orbitrap mass spectrometers, made a change. A resolution of ≥ 120.000 and accurate masses with mass errors below 1 ppm do now allow full scan detection of trace amounts of single compounds even in high matrix samples with ion chromatogram extraction at accurate masses [20].

High-resolution hybrid mass spectrometers using an additional quadrupole (HRQTOF; HRQ-Orbitrap) offer the possibility to combine high-resolution power and the selectivity of a targeted measurement (MS/MS). This results in an enhanced selectivity and helps to distinguish isomeric compounds often occurring in natural samples, or to characterize individual classes of analytes [21]. Via parallel reaction monitoring (PRM), information analogous to that from SRM on a QqQMS can be obtained on an HRQ-Orbitrap-MS.

There are some rare reports comparing QqQ with HRMS, such as for the analysis of drugs in plasma samples [22], polyphenols in wine [23], or drugs in honey and animal tissue [24], but so far no studies on B vitamins have been performed. All three publications concluded the tested QqQ and Orbitrap mass spectrometers to give very similar results with respect to peak shape, limits of quantification, and linearity. Cavaliere et al. (2019) found that the Q Exactive hybrid mass spectrometer, operated in a type of PRM, was even superior to the QqQMS [23]. All three approaches underline the great potential offered by HRMS due to the unpretentious operation and higher information content of full scan measurements [22–24].

**Fig. 1:**
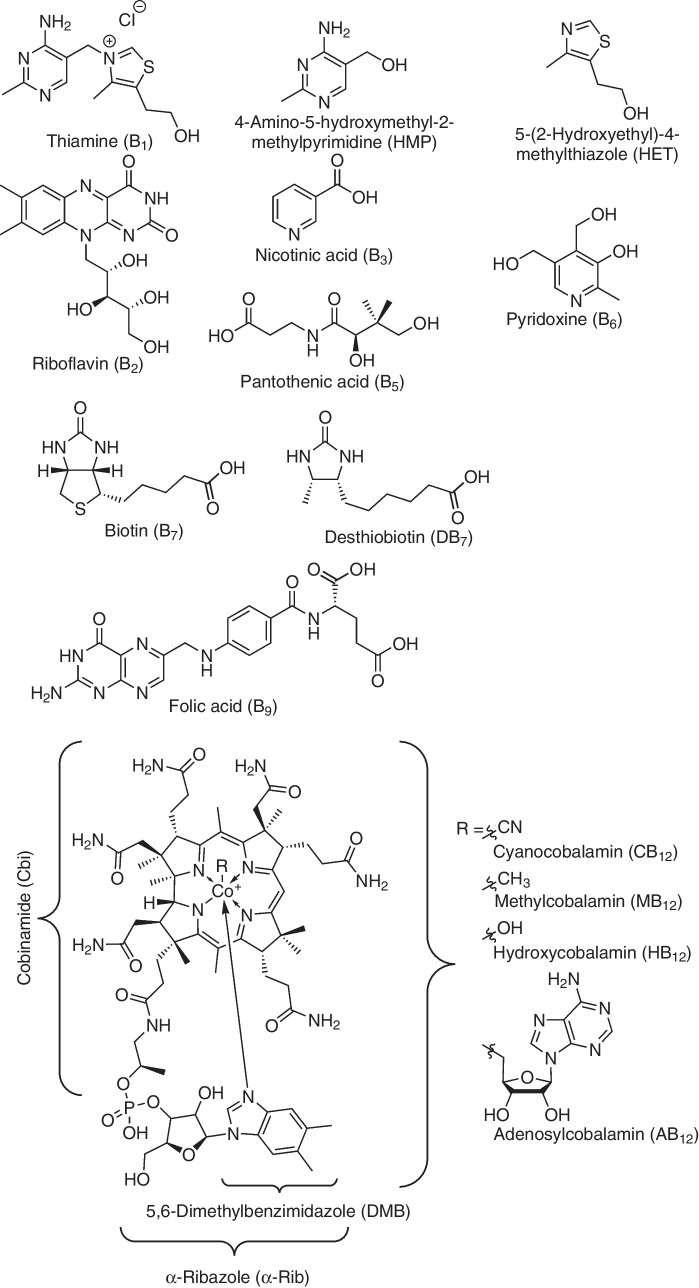
Chemical structures of the studied B vitamins and selected biosynthetic precursors

In this paper, we present the development of a method for the simultaneous measurement of all eleven B vitamins (thiamine (B_1_), riboflavin (B_2_), nicotinic acid (B_3_), pantothenic acid (B_5_), pyridoxine (B_6_), biotin (B_7_), folic acid (B_9_), cyanocobalamin (CB_12_), adenosylcobalamin (AB_12_), methylcobalamin (MB_12_), and hydroxycobalamin (HB_12_)) including five biosynthetic precursors (two B_1_ precursors: 4-amino-5-hydroxymethyl-2-methylpyrimidine (HMP) and 5-(2-hydroxyethyl)-4-methylthiazole (HET); a B_7_ precursor: desthiobiotin (DB_7_); and two B_12_ precursors: 5,6-dimethylbenzimidazole (DMB), and cyanocobinamide (Cbi)) (Fig. 1) at trace levels on a QqQMS in SRM mode and on a Q-Orbitrap in PRM and full scan modes. The different methods are thoroughly compared in terms of their limits of detection (LOD), linear range of calibration, and the influence of matrix effects on the respective characteristic quantitation properties/parameters and applied to a seawater sample and to the cell extract of a bacterial culture.

## Material and methods

### Chemicals

Standard compounds were purchased from Sigma-Aldrich (Steinheim, Germany) in analytical purity. α-Ribazole was synthesized according to Wienhausen et al. [25]. Ultrapure water was obtained by filtration of deionized water in an arium® pro ultrapure water system (Sartorius, Göttingen, Germany). Acetonitrile and methanol were purchased from Biosolve (Dieuze, France) in ULC/MS grade.

### Standard compound solutions

Stock solutions of 200 µg/mL in ultrapure water were prepared for each standard compound. Only vitamins B_7_ and B_9_ were dissolved after addition of 1% of an aqueous ammonium hydroxide solution (25% NH_4_OH_aq_), and B_2_ after addition of 5% acetonitrile for better solubility. The stock solutions were diluted to 20 µg/mL with ultrapure water. Mixed standard compound solutions were prepared from 20 µg/mL stock solutions of individual standard compounds with different concentrations for each vitamin according to their expected concentration ranges (ESM Table S1).

### LC/MS instruments and chromatography

All technical equipment used came from Thermo Fisher Scientific (San Jose, CA, USA). A TSQ Quantum UltraAM triple quadrupole mass spectrometer was used in connection with a heated electrospray ionization source (HESI-II). The coupled HPLC was an Ultimate 3000 HPLC with an LPG 3400 MB pump, a cooled WPS-3000TSL Micro auto sampler set to 7 °C, and a TCC-3200 column oven set to 35 °C. For high-resolution measurements, an Orbitrap Fusion, with an EASY-Max NG source equipped with a HESI-II probe, was used. Here, chromatographic separation was achieved using a Vanquish Flex UHPLC with a VF-P10-A pump, a VF-A10-A auto sampler set to 7 °C, and a VH-C10-A column oven set to 35 °C.

Chromatography was performed on a Kinetex Evo C18 column (150 × 2 mm, 2.6 µm, Phenomenex, Torrance, CA, USA). 5 µl of sample were injected for each measurement, and the solvent flow rate was 400 µl/min. 10 mM ammonium formate with a pH of 6.0 (eluent A) and acetonitrile (eluent B) were used, and the solvent gradient was as follows: 0–13 min 100–75% A; 13–15 min 75–0% A; 15–19 min 0% A; 19–21 min 0–100% A; 21–26 min 100% A.

**Fig. 2:**
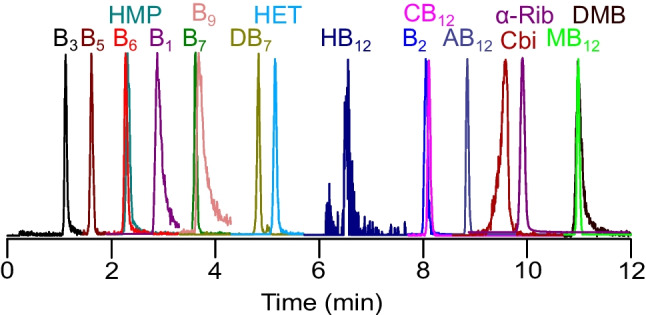
Chromatographic separation of 17 different vitamins and biosynthetic precursors at pH 6.0, measured with SRM represented by the individual extracted ion chromatograms (EIC)

The applied solvent gradient resulted in satisfactory separation of the 17 analytes, with only isolated peak overlap of a maximum of two signals (Fig. 2). Apart from the improved clarity of the chromatograms, good separation has the advantage that for SRM measurements fewer components have to be combined into individual time separated scan segments and more scans per analyte signal are obtained.

### Optimization of the source parameters for electrospray ionization

#### TSQ

To determine the optimal source parameters, each analyte was dissolved in ultrapure water and injected directly into the mass spectrometer via a syringe pump at a concentration of 20 µg/mL. The intensity of each molecular ion and the influence exerted by the variation of different source parameters were monitored. Different values for spray voltage (± 2–4 kV), vaporizer temperature (200–450 °C), capillary temperature (280–340 °C), sheath gas pressure (20–80 arbitrary units), auxiliary gas pressure (0–20 arbitrary units), and tube lens voltage (0–240 V) were tested on the TSQ in positive as well as in negative ionization mode.

#### Orbitrap

On the Orbitrap, the values for spray voltage (± 2–5.5 kV), vaporizer temperature (280–400 °C), transfer tube temperature (250–350 °C), sheath gas pressure (20–80 arbitrary units), auxiliary gas pressure (0–25 arbitrary units), and RF lens voltage (0–120 V) were varied and tested in positive and negative ionization mode. The final, optimal conditions for each vitamin are listed in ESM Table S2.

### MS parameter optimization

#### TSQ

On the TSQ, the vitamins were measured in SRM. To develop the method, the intensities and masses of resulting fragment ions were examined by varying the collision energy (0–70 eV) during direct injection of each vitamin standard. The collision gas was argon (0.0024 hPa). Fragment ions and associated collision energies most suitable in terms of intensity and selectivity were used for SRM measurement (Table 1). Experiments for the optimization of the scan time (0.01–0.5 s), scan width (0.3–2 atomic mass units (amu)), and number of fragment ions (2 or 3) were performed.

**Table 1:** Parameters for the full scan and PRM method at the Orbitrap Fusion and for the SRM method at the TSQ Quantum

		Orbitrap		TSQ SRM	
Vitamin	Chemical formula	Parent ion *m/z* → product ion *m/z* (collision energy eV)	RF lens (V)	Parent ion *m/z* → product ions *m/z* (collision energy eV)	Tube lens (V)
Thiamine (B_1_)	C_12_H_17_N_4_OS	**265.1118** → 122.0731 (22)	50	**265.1** → *122.1* (20), 144.1 (18), 81.1 (40)	112
Riboflavin (B_2_)	C_17_H_20_N_4_O_6_	**377.1456** → 243.0875 (30)	90	**377.1** → *243.0* (32), 172.1 (49), 198.0 (50)	126
Nicotinic acid (B_3_)	C_6_H_5_NO_2_	**124.0393** → 96.0444 (50)	50	**124.0** → 80.1 (24), *78.1* (25), 53.2 (32)	110
Pantothenic acid (B_5_)	C_9_H_17_NO_5_	**220.1179** → 90.0550 (25)	50	**220.1** → 90.1 (20), *202.1* (17), 184.1 (16)	108
Pyridoxine (B_6_)	C_8_H_11_NO_3_	**170.0812** → 152.0716 (20)	60	**170.1** → *152.1* (18), 134.1 (28), 77.1 (46)	96
Biotin (B_7_)	C_10_H_16_N_2_O_3_S	**245.0954** → 227.0847 (24)	55	**245.1** → *227.1* (19), 97.1 (41), 123.1 (38)	110
Folic acid (B_9_)	C_19_H_19_N_7_O_6_	**442.1470** → 295.0936 (18)	50	**442.1** → *295.1* (23), 176.0 (51), 199.9 (49)	165
Cyanocobalamin (CB_12_)	C_63_H_88_CoN_14_O_14_P	**678.2910** → 147.0917 (50)	60	**678.4** → 358.1 (43), 997.5 (39), *147.0* (57)	135
Adenosylcobalamin (AB_12_)	C_72_H_100_CoN_18_O_17_P	**790.3364** → 665.2893 (16)	60	**790.5** → 665.0 (39), *147.0* (59), 358.9 (54)	153
Methylcobalamin (MB_12_)	C_63_H_91_CoN_13_O_14_P	**672.8012** → 665.2892 (17)	60	**672.9** → 665.3 (33), *146.9* (57), 971.5 (54)	138
Hydroxycobalamin (HB_12_)	C_62_H_89_CoN_13_O_15_P	**673.7908** → 147.0917 (49)	60	**673.8** → *664.9* (10), 635.7 (33), 147.0 (53)	170
4-Amino-5-hydroxymethyl-2-methylpyrimidine (HMP)	C_6_H_9_N_3_O	**140.0818** → 81.0448 (35)	60	**140.1** → *81.1* (24), 122.1 (16), 54.2 (37)	81
5-(2-Hydroxyethyl)-4-methylthiazole (HET)	C_6_H_9_NOS	**144.0478** → 113.0294 (48)	65	**144.1** → *113.1* (29), 126.1 (23), 71.1 (47)	82
Desthiobiotin (DB_7_)	C_10_H_18_N_2_O_3_	**215.1390** → 197.1283 (20)	60	**215.1** → *197.2* (17), 179.2 (23), 95.1 (32)	97
5,6-Dimethylbenzimidazole (DMB)	C_9_H_10_N_2_	**147.0917** → 131.0604 (55)	70	**147.1** → *131.1* (43), 77.1 (55), 120.1 (29)	82
Cyanocobinamide (Cbi)	C_49_H_72_CoN_12_O_8_	**1015.4923** → 930.4505 (35)	70	**1015.6** → *930.4* (68), 1014.2 (68), 988.6 (39)	232
α-Ribazole (α-Rib)	C_14_H_18_N_2_O_4_	**279.1339** → 147.0917 (40)	60	**279.1** → 132.1 (58), *147.1* (22)	93

Bold numbers, parent ion *m/z*; italic numbers, most intense SRM product ion *m/z* used for quantification

#### Orbitrap

Although performed on an Orbitrap Fusion, the methods presented are also compatible with the more common Q Exactive. For detecting trace concentrations of B vitamins, two different measurement methods, a PRM and a full scan method, were developed and compared. For the PRM method, in analogy to the SRM method applied on the TSQ, a list of molecular masses, their adduct ions, charges, and corresponding retention time windows was generated. Values for the higher energy collisional dissociation (HCD) energy, RF lens, and scan range were also included in the list. The Orbitrap scan range in PRM was always *m/z* 50–500, except for the cobalamins and Cbi, where it was *m/z* 110–1100. All values were optimized via direct injection for all vitamins and precursors to establish the most intense fragment ion in an MS/MS experiment. By varying the quadrupole isolation window (1–3.6 amu), resolution (15.000–120.000), maximum injection time (30–100 ms) to fill the Orbitrap mass analyzer, and the average gain control (AGC) target (1.0E4–1.0E6), which controls the number of ions that enter the Orbitrap, the best values for the analytes studied were evaluated.

In full scan mode, based on the optimized values for the PRM method, the *m/z* range of 110–1100 was scanned in the Orbitrap analyzer with a resolution of 120.000. The RF lens was set to 60 V, the AGC target to fill the C-trap was 4.0E5, and the maximum injection time was 50 ms. Spectra were acquired in centroid mode and calibrated with the instrument’s internal calibration substance fluoranthene via Easy-IC.

### Dilution series

The mixed standard compound solution used for the optimization experiments was diluted in several steps with ultrapure water to obtain a concentration series ranging from 150–0.5 to 600.000–2000 ng/L. Same concentration series were used to examine the analytical limits of the different measurement methods on both instruments.

### Impact of seawater matrix on signal responses

Representative for a marine matrix sample, seawater from the Wilhelmshaven marina (53°30′54″N, 8°9′0″O, North Sea, Germany) was collected in December 2020 using a stainless-steel bucket, filtered (0.22 µm, 47 mm, mixed cellulose ester, Merck, Darmstadt, Germany), and stored in the dark at 4 °C until further processing. Solid phase extraction on a PPL cartridge at pH 6 was used to concentrate 1000 mL of seawater sample down to 0.2 mL extract. All extracts were measured in triplicates, and the vitamin contents were estimated using an external calibration line. Based on these contents, aliquots of the extract were spiked with approximately twice, five times, and ten times the amount of vitamins, and measured. In addition, ultrapure water samples without matrix were spiked with the same amounts of vitamins as the extract aliquots and measured in triplicates as well. This resulted in two calibration lines, one with the influence of matrix on signal intensity and one without, which were compared. The difference in slope of the two calibration lines can be used to define the matrix-induced ion suppression. By extrapolating the standard addition calibration line of the seawater sample to the *x*-axis, the content of vitamins in the sample can be determined. The added standard compounds experience the same suppression as the analytes in the sample and thus, the value of the *x*-axis represents the analyte concentration under consideration of matrix.

### Culture conditions for intracellular bacterial vitamin determination

*Phaeobacter inhibens* (DSM 17395), a model organism of the Roseobacter group, was grown in artificial seawater (ASW) medium (pH 8) in a volume of 400 mL in 1000 mL Erlenmeyer glass flasks. Besides the addition of glucose (5 mM) as the sole source of carbon and energy, no other organic compounds were added to the medium. Bacteria were cultivated in triplicates at 20 °C in the dark on a shaker (100 rpm). A sterile flask with ASW-medium supplemented with glucose (5 mM) was run as negative control. Samples for bacterial cell counts and intracellular bacterial vitamin measurement were withdrawn under laminar flow. Bacterial cells were fixed with 2% glutaraldehyde, incubated at 4 °C for 30 min, and frozen at − 20 °C until enumerated by flow cytometry (BD Biosciences Accurri C6), as previously described [26]. Bacterial biomass for intracellular vitamin determination was collected by centrifugation (7000 rpm, 7 min). The supernatant was discarded and cell pellets were frozen at − 20 °C for further analysis.

### Extraction of intracellular vitamins

Sample preparation for analysis of intracellular vitamin B content in bacteria was performed using a slightly modified version of the method described for the analysis of bacterial coenzyme a thioesters [21]. Frozen cell pellets were resuspended in 1 mL of methanol, transferred into 2 mL polypropylene tubes (Sarstedt) containing 0.5 mL glass beads (0.7 and 0.1 mm, Carl Roth), and broken up by homogenization in a mini-bead beater (biospec products). In addition, the extraction was repeated twice with 0.5 mL of methanol, and the solvent was evaporated under a gentle flow of nitrogen gas. All other steps were performed according to Cakić et al. [21]. The filtered solution was analyzed by liquid chromatography coupled with mass spectrometry. Bacterial vitamin concentrations were calculated by external quantification, as previous tests for recovery and matrix effect have shown that the analytes were all recovered at 98–100% with no ion suppression.

### Evaluation

Data evaluation was performed using the Thermo Scientific Xcalibur software version 4.2.28.14 on the Orbitrap and version 4.1.50 on the TSQ. For the SRM measurements on the TSQ as well as the PRM measurements on the Orbitrap Fusion, the extracted ion chromatograms (EIC) of the most intense fragment ions (± 0.1 amu on the TSQ and ± 20 ppm on the Orbitrap) of the respective analytes were chosen. For full scan measurements on the Orbitrap, the EIC of the respective molecular ion (± 5 ppm) was observed. Areas and general parameters of resulting signals, such as scans per peak and signal to noise (S/N) ratio, were chosen to compare the respective approaches. Blanks were measured after every sample and every standard compound solution to ensure that no carry-over effects occurred. Intra-day (*n* = 5) and inter-day (*n* = 5) precision have been evaluated by measuring standard compounds multiple times over the course of 5 days and resulted in less than ± 10% variation for every analyte.

## Results and discussion

### Optimization of the source parameters for electrospray ionization at the TSQ and the Orbitrap

Since the sources of the two mass spectrometers are of the same type (HESI-II), it is not surprising that all vitamins are optimally ionized under similar conditions (ESM Table S2). With respect to the analytes, it became apparent that higher capillary and vaporizer temperatures had a positive effect on the signal regarding shape, intensity, and S/N ratios, due to the improved solvent evaporation. Low sheath gas flows led to unstable signals that showed high fluctuations in intensity. The influence of auxiliary gas flow was less pronounced. Most of the analytes were if at all only slightly affected by the spray voltage and showed no strong variation with respect to their intensity on both mass spectrometers. Optimal values for tube lens voltage at the TSQ were individual for each analyte and ranged from 81 to 232 V. Larger molecules required a higher tube lens voltage for optimal sensitivity. The RF lens voltages at the Orbitrap were mostly 60–70 V, with the best values for the B_12_ vitamins and Cbi being up to 120 V (Table 1).

Irrespective of the mass spectrometer type, experiments performed in negative ionization mode showed less intense signals than in positive mode for the given vitamins. In addition, the presence of many interfering background ions from e.g. the solvents resulted in poorer S/N ratios in negative ionization mode. In the case of B_3_, HMP, HET, Cbi, DMB, and the B_12_ vitamins, the target compounds were not even visible. Consequently, all experiments were performed in positive ESI mode.

### MS parameter optimization

The respective precursor and product masses as well as the collision energies at which the fragment ions were most intense were determined manually. The results of these experiments are summarized in Table 1. B_1_ and Cbi show no adduct formation and appear as [M]^+^, only. All other analytes appear as protonated species [M + H]^+^. Except the four cobalamins that appeared double charged, all other ions were single charged.

#### TSQ

**Fig. 3:**
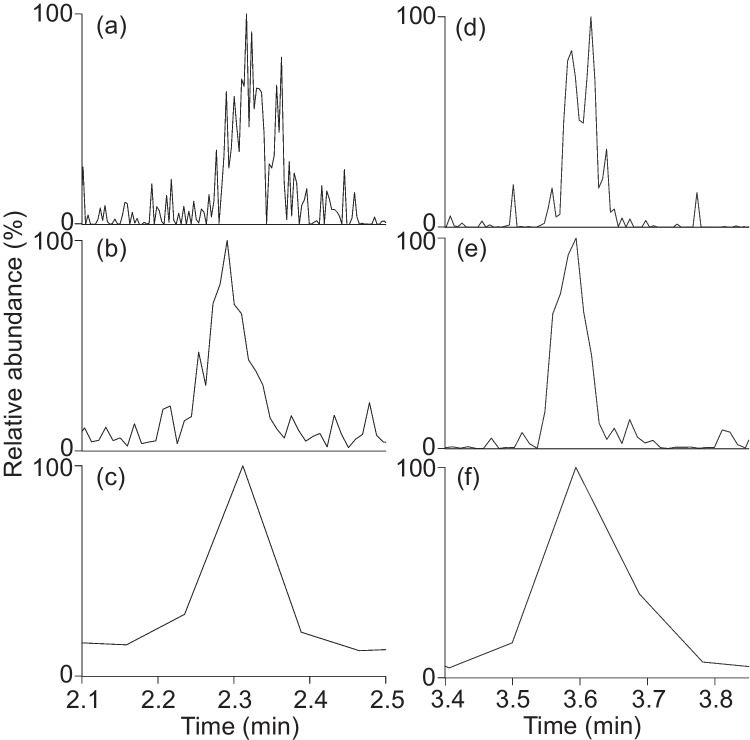
Signals from B_6_ (left) and B_7_ (right) of SRM (TSQ) measurements with scan times of **a** + **d** 0.01 s, **b** + **e** 0.05 s, and **c** + **f** 0.5 s

To enhance the detection selectivity and sensitivity, up to three fragment ions per analyte were selected for the SRM method. Some B vitamins (e.g., B_1_, B_2_, B_6_, B_7_, and DB_7_) fragmented almost completely into one or two characteristic and intense fragment ions under MS/MS conditions and facilitated the selection of suitable product masses for quantification. However, some analytes fragmented at a given collision energy into several fragment ions of moderate intensity, or insufficiently, leaving the intact molecular ion (ESM Fig. S1). Here, the selection of optimal fragment ions was rather difficult and often resulted in reduced sensitivities. In particular, since molecular ions are excluded from Q3 detection and do not contribute to signal formation, a loss of signal intensity is to be expected with incomplete fragmentation. Scan times of 0.05 s (or 0.075 s for the B_12_ vitamins) per fragment ion turned out to be the preferable value. An extended scan time of 0.2 s resulted in signals based on three scans for some compounds, which is not reasonable for peak reconstruction. In contrast, scan times considerably below 0.02 s tend to result in split signals that require peak smoothing and risk a distortion of the resulting estimated area. Figure 3 shows the results for B_6_ and B_7_ as representative examples.

The optimal scan width was set to ± 0.3 amu. This avoided interfering ions being passed through the filter quadrupole and deteriorate the quality of the chromatogram by increasing the background. Additionally, the narrow scan width improved the S/N ratios and signal areas. Caution was required for the B_12_ vitamins and Cbi. Since their fragment ions are larger, the given mass accuracy of the TSQ is lower. Consequently, if the scan width is too narrow, the masses might partially miss the selected window. For these compounds, a scan width of 1.0 amu was reasonable, as in the samples studied, the abundance of potentially interfering ions above *m/z* 600 was low (ESM Fig. S2).

#### Orbitrap PRM

During the PRM method run on the Orbitrap Fusion, the molecular ions of the individual analytes were selected and isolated in the quadrupole. For quadrupole isolation, better peak shape and larger peak areas were obtained with a larger isolation window, but it needs to be considered that the selectivity of individual analytes decreases with large quadrupole isolation for natural samples. Since analytes other than the observed vitamins and precursors may pass through the mass filter, an optimal isolation window of 3.0 amu was determined. After fragmentation (HCD) in the ion routing multipole, the fragment ions were analyzed in the Orbitrap. Via direct injection, the most intense fragments of each analyte were determined in advance with the collision energy as well as appropriate values for the RF lens, chosen in preliminary experiments. These respective initial data are summarized in Table 1. In this context, various MS parameters of the Orbitrap Fusion were also tested. For standard compound solutions, some parameters did not have any noticeable effect on signal quality, such as the AGC target, which was set at 1.5E5. However, if matrix-rich natural extracts with low analyte concentrations are measured, the AGC target becomes relevant. It controls the number of ions entering the Orbitrap to prevent the trap from being overloaded, but on the other hand, it can also cause the analyte signal to be reduced or disappear completely, when other compounds of similar molecular mass and retention time with higher concentration are present in the sample. The resolution has a distinct effect on the signal quality. At low resolution of 15.000, some of the peaks had a jagged form because of low mass accuracy, whereas at high resolution of 120.000, the S/N ratio deteriorated because of increased scan time, while the peak shape improved for other signals (ESM Fig. S3). For our experiments, a resolution of 60.000 proved to be the best choice for all analytes. Similarly, for the injection time, the peak area and peak shape improved with higher values, at the cost of the S/N ratio. For the final method, an injection time of 100 ms was chosen. However, it must be kept in mind that depending on which condition is met first for a sample, either the AGC target or the injection time is limiting.

#### Orbitrap full scan

Overall, the full scan mode allows almost no analyte-specific optimization options. Since the RF lens in full scan cannot be set individually, one fixed value for the whole measurement is necessary as a compromise. For individual analytes, RF lens values were all in the range of 50 to 70 V, so 60 V was chosen as the value. Compared to the PRM method, the AGC target was increased to 4.0E5 and the maximum injection time was reduced to 50 ms, since without prior quadrupole selection of the masses in full scan, more ions have to be analyzed in a shorter time. In addition, the resolution was increased to 120.000 for the full scan measurements. This supported the subsequent identification of the searched molecular masses.

### Trace-analytical potential of the three different measurement methods

**Fig. 4:**
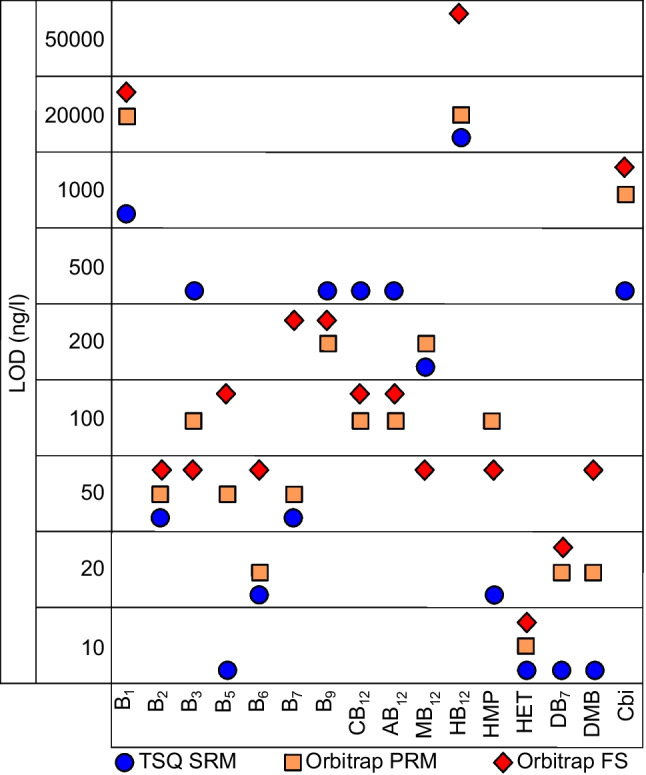
Instrumental LOD in ng/L for SRM (circle, blue), PRM (square, yellow), and full scan (diamond, red). Data represent the lowest concentration that gave a reproducible signal in the linear range

The individual LOD and linear ranges were determined with a standard compound dilution series. The results are given in Fig. 4 and Table 2, and ESM Table S3 (for LOD in ng/L and pg on column).

**Table 2:**
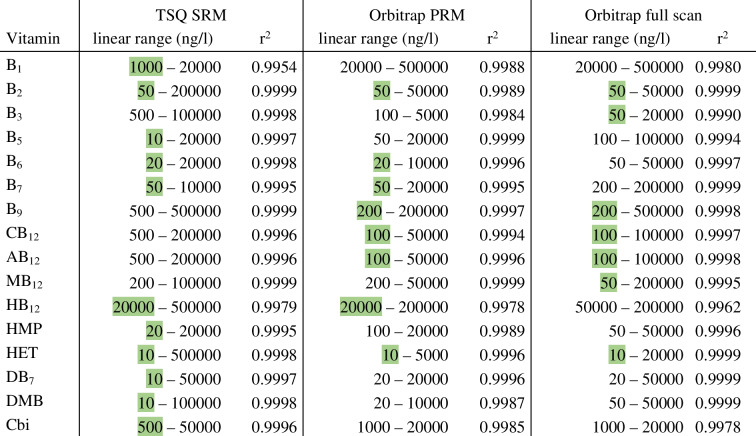
Linear range of each vitamin and regression coefficients of the corresponding linear regression. Lowest limits of detection are shaded

A common measure to determine LOD and LOQ is based on the S/N ratio of given concentrations. The LOD corresponds to S/N 3 and the LOQ to S/N 10, respectively. However, when EIC are used, particularly if high-resolution data are processed, often mass selectivity suppresses any noise [27] and disables a meaningful S/N calculation of a detected signal (ESM Fig. S4). Alternatively, the lowest detectable concentration of the dilution series within the linear range was given as an equivalent for the LOD in this study (Table 2). Accordingly, it can be assumed that real LODs are even lower.

With exceptions of B_1_ and HB_12_ that stood out with comparable low sensitivity, the lowest detectable concentrations range between 10 and 500 ng/L, equivalent to 0.05–2.50 pg on column absolute (ESM Table S3).

The comparison of SRM, PRM, and full scan modes, shown in Fig. 4, resulted in the TSQ in SRM mode to give the lowest detectable concentrations for vitamins B_1_, B_5_, HMP, DB_7_, DMB, and Cbi. The PRM method (Orbitrap) was not superior with respect to detection sensitivity. With SRM and PRM, the lowest detectable concentrations for B_6_, B_7_, and HB_12_ were achieved. For B_3_ and MB_12_, the lowest detectable concentrations have been reached with the full scan method on the Orbitrap, while both the PRM and full scan measurement reached the lowest detectable concentrations for B_9_, CB_12_, and AB_12_. All three measurement methods detected the lowest concentrations for B_2_ and HET.

In most cases, the lowest detectable concentrations of the respective vitamins for the three measurement modes are highly comparable. They differ by a factor of up to five only for most vitamins, thus it is not possible to classify one method as clearly superior to the others. Only for B_1_, B_3_, and B_5_ did the detection limits differ by a factor of 10 or 20, depending on the measurement method. The lowest detectable concentrations ranged between 10 and 200 ng/L for most of the analytes and represent trace levels. Only B_1_, HB_12_, and Cbi showed less sensitivity above 500 ng/L for all three methods.

Irrespective of the tested methods, the instrumental detection limits achieved in this study are lower than those reported in previous studies [2, 12, 15, 28, 29] for B_2_, B_5_, B_7_, CB_12_, AB_12_, MB_12_, and HMP. Except for B_1_, B_9_, HB_12_, and Cbi, all other analytes showed signals at concentrations below 200 ng/L. All individual calibrations show linear behavior between two and four orders of magnitude, with good regression coefficients (*r*^2^ > 0.995) (Table 2). To our knowledge, instrumental linear calibration ranges including concentrations much lower than 1000 ng/L have not been reported before. We thus conclude that the approaches developed in this study represent an improvement of sensitivity with respect to so far published data [1, 2, 15, 28].

Although the detection limits of the B vitamins achieved are very low, they are still insufficient for the direct measurement of these target compounds in seawater. At least a pre-concentration factor of ten to a thousand, depending on the analyte, is inevitable.

### Effect of natural seawater matrix on the methods’ performances

A natural seawater sample was used to investigate to what extent matrix of a natural sample has an influence on the retention time and signal intensity of vitamins, in comparison with standard samples in ultrapure water.

Almost all of the chemically very heterogenous vitamins and precursors included in this study were detected in the solid phase-extracted seawater sample with all three tested methodological approaches (ESM Fig. S5).

To prove the effect of natural seawater matrix on the retention behavior of the studied compounds, the resulting chromatogram of an ultrapure water sample spiked with the analytes of interest was compared with the (already mentioned) seawater extract. For most vitamins and their precursors, the retention behavior was not affected. It is normal that small retention time shifts occur occasionally in LC–MS. However, B_1_, B_5_, B_7_, and HMP showed elongated retention times of more than 0.3 min when measured in seawater matrix (ESM Table S4). Even though this retention time shift is relatively small, it is noticeable that the shifted analytes would elute successively with similar retention times under standard conditions. In the presence of matrix, other constituents probably elute at this retention time with corresponding polarity that interact with the analytes and thus change the interaction with the solid phase of the column, which can lead to a deviating retention time. Similar shifts were observed in a study of matrix effects on the retention behavior of bile acids from pig urine [30]. Consequently, predefined retention time windows typical for SRM/PRM methods might miss signals and lead to a false negative result. With this in mind, a full scan method is definitely advantageous.

**Fig. 5:**
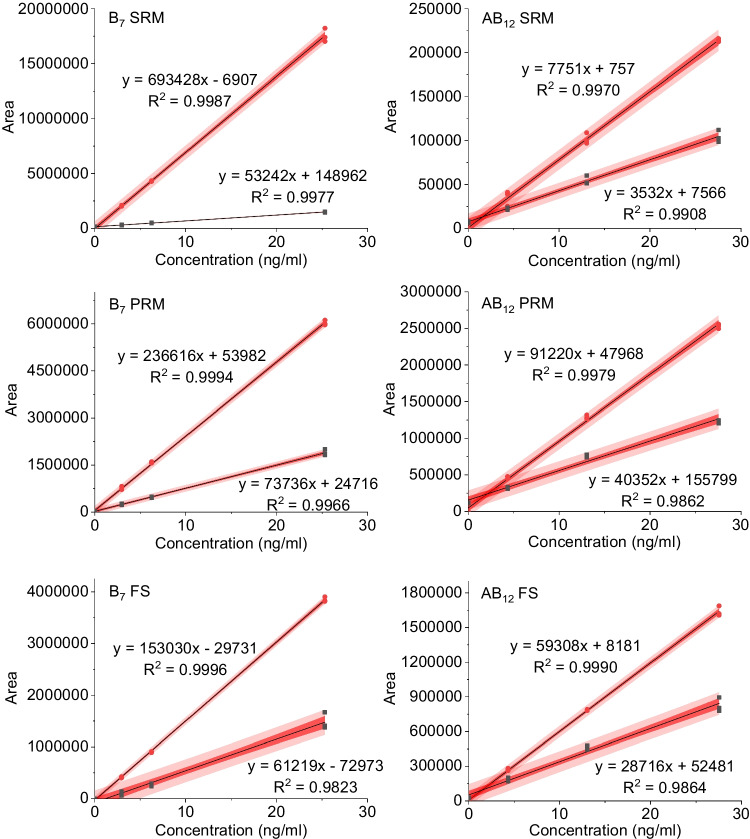
Regression lines illustrating linear response and calibration behavior of the respective analytes. Furthermore, ion suppression via addition of known amounts of B_7_ (left) and AB_12_ (right) to ultrapure water (red circles) and a processed seawater sample (black squares) is reflected by the decline in slope. The three methodological approaches compared are SRM on the TSQ Quantum, PRM and full scan (FS) on the Orbitrap Fusion, respectively. The regression lines in black with the linear equations and the squared correlation coefficients are displayed as well as the 95% confidence (dark red) and prediction (light red) bands

Any matrix effect on ESI signal suppression or amplification of the respective compounds was studied on spiked pre-concentrated seawater samples. For this purpose, they were amended with the respective analytes at different concentration, directly comparable with those used for calibration. The areas of the signals generated by the standard addition method were exemplarily plotted against the concentration of each analyte and the differences in the equations obtained by linear regression of the resulting curves were compared (Fig. 5 and ESM Fig. S6–S12). A decrease in slope in the presence of matrix is a clear sign of matrix-induced ion suppression.

**Fig. 6:**
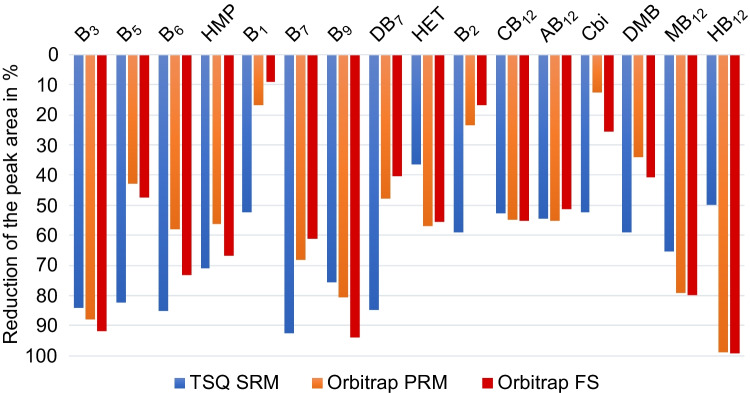
Reduction of the peak area of each vitamin and precursor (from left to right in the order of elution) in the presence of matrix compared to ultrapure water for the three MS methods (selected reaction monitoring (SRM), parallel reaction monitoring (PRM), and full scan (FS)) to visualize the ion suppression

To highlight differences in the observed suppression in-between the applied methods, the difference was expressed in relative reduction of the peak area of a signal in the presence of natural matrix in comparison to ultrapure water (Fig. 6) which functions as a representative indicator of matrix-induced ion suppression. Ion suppression is mainly an effect of the ion source. The sources of the two mass spectrometers are of the same type, but differ in the year of construction, which can be seen from the fact that the values for PRM and full scan at the Orbitrap are mostly comparable. The matrix effect at the TSQ differs partially from that of the Orbitrap. The optimal source parameters for vitamin analysis, such as vaporizer and transfer tube temperature and spray voltage, are not exactly the same for the two mass spectrometers. As a result, the same matrix components may be ionized at different efficiencies and individual vitamins may experience different levels of competition in ionization, potentially resulting in higher ion suppression of an analyte on one mass spectrometer than on the other. Disregarding the methodological approach applied, residual matrix from marine water had a strong effect on the signals of vitamins. Losses in intensity were observed between 10 and more than 90%. Analytes eluting early showed a stronger ion suppression than the late-eluting vitamins (Fig. 6), which is an indication that ion suppression strongly depends on the retention time and thus on the elution of other matrix constituents.

The observed ion suppression could be due to the fact that at the beginning of the measurement, a large proportion of polar matrix compounds is transported into the ion source with the aqueous eluent as well. Competitive reactions and interactions might reduce the intensity of the polar analytes eluting at the same time.

Interfering compounds compete or interact with the analytes in the ion source in different ways. In some cases, they may be present at the surface of the droplets during their formation in the ESI source and prevent the analytes from reaching the surface, so that they cannot leave the droplet as charged ions as the solvent evaporates. Furthermore, neutral molecules with high proton affinity may neutralize analyte molecules already ionized in the source by proton transfer, such that they do not contribute to signal formation, which leads to a reduction of the resulting peak [31].

In Fig. 5, the examples of the earlier eluting B_7_ and the later eluting AB_12_ show that the slope for B_7_ in the presence of matrix is much lower than for AB_12_ for all three methods, thus B_7_ experiences stronger ion suppression, which therefore appears to be dependent on retention time and thus on the presence of different eluting matrices. Additionally, B_7_ shows a relatively large shift in its retention time, depending on the LC–MS-system, if matrix is involved. This leads to a small but noticeable increase in the ion suppression for the SRM method on the HPLC-TSQ-system, because the retention time shift is larger than on the UPLC-Orbitrap-device.

Although the measurement was preceded by extensive sample purification (SPE) and numerous matrix constituents were removed, the observed ion suppression is quite pronounced, as many compounds with a polarity comparable to that of the vitamins are still present and were probably also concentrated. Accordingly, external calibration and quantification without consideration of matrix would result in large underestimations of given concentrations and substantial analytical errors. If ion suppression is observed, isotopically labeled internal standard compounds are used to compensate matrix effects in trace quantification, which is a common, effective but cost intensive solution. By adding such standard compounds, which chemically behave almost identically to the unlabeled compounds, and using these standard compounds for quantification, any effects are compensated. Besides the cost, a major disadvantage is that not all analytes considered in this work are available isotopically labeled [31], in particular various precursors and B_12_ variants studied here. Due to the heterogeneous polarity of the B vitamins, the use of only selected labeled standard compounds is no solution. Alternatively, as performed in our study, a concentration series of regular standard compounds in the appropriate matrix can be applied (calibration via standard compound addition). The corresponding inverse calibration enables a reliable quantification.

Based on this procedure, the B vitamins and precursors of the examined seawater sample from the North Sea were determined (Table 3 and ESM Table S5 for pico molar (pM) concentrations). Again, all measurements were performed with the three different methods SRM, PRM, and full scan. With respect to the basically very low compound concentrations in marine water and accordingly associated difficulty of reliable quantification, the generated results are highly convincing and range in the same order of magnitude for the respective compounds for all three methods. This underlines the applicability of any of these presented methods as suitable for the simultaneous trace detection and quantification of all B vitamins in seawater samples.

**Table 3:** Vitamin content in a seawater sample from the North Sea in ng/L

Vitamin	SRM (ng/L)	PRM (ng/L)	Full scan (ng/L)
B_1_	7.1 ± 2.0	4.4 ± 1.4	nd
B_2_	13.5 ± 0.9	12.0 ± 1.3	7.0 ± 0.3
B_3_	nd	3.2 ± 0.5	5.7 ± 1.2
B_5_	21.3 ± 1.8	11.7 ± 0.7	8.5 ± 1.0
B_6_	6.8 ± 2.1	5.3 ± 0.4	15.4 ± 1.5
B_7_	7.3 ± 0.6	0.2 ± 0.2	nd
B_9_	nd	nd	nd
CB_12_	20.9 ± 2.3	29.9 ± 3.4	20.9 ± 1.6
AB_12_	0.9 ± 0.2	1.7 ± 0.3	0.7 ± 0.3
MB_12_	nd	3.1 ± 1.8	nd
HB_12_	nd	nd	nd
HMP	2.2 ± 0.3	1.8 ± 0.2	3.8 ± 0.6
HET	0.2 ± 0.0	0.4 ± 0.0	0.2 ± 0.0
DB_7_	1.2 ± 0.1	0.3 ± 0.0	nd
DMB	2.0 ± 0.1	2.0 ± 0.3	1.4 ± 0.1
Cbi	1.1 ± 0.2	0.9 ± 0.1	0.7 ± 0.4

*nd*, not detected

The presented quantitation principle allows reliable quantification but does not provide any information on the losses during the workup. For this purpose, a natural sample can be divided into several aliquots and mixed with different amounts of analyte. These samples are processed and measured identically. By plotting the resulting peak areas against the amount of analyte added, a calibration line is obtained which reflects the loss during the processing as well as the influence of matrix. Although this method is very accurate, it is also extremely time-consuming [31].

**Fig. 7:**
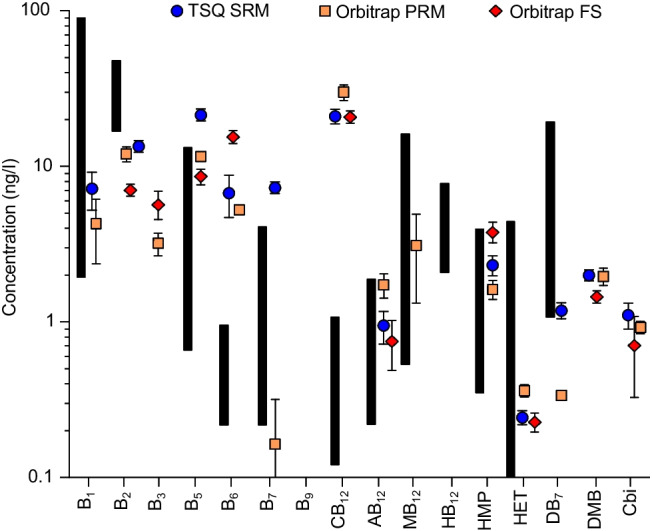
Vitamin concentrations detected by SRM (circle, blue), PRM (square, yellow), and full scan (diamond, red) in the North Sea compared to literature values (bars) found by Heal et al. [11] in the Hood Canal (B_2_, B_6_, B_7_, AB_12_, HB_12_), Suffridge et al. [10, 12] in the Eastern and Central Atlantic Ocean (B_1_, CB_12_, MB_12_, HMP, HET), and Longnecker et al. [14] in the Eastern Pacific Ocean (B_5_, DB_7_)

In Fig. 7, the vitamin concentrations found in the North Sea water sample are compared with available literature values from seawater samples. Although the samples derive from different locations, these literature values fall in the same order of magnitude for most vitamins. The largest deviations to higher concentrations with respect to the North Sea water were observed for B_6_ and CB_12_. This might be related to higher nutrient concentrations compared to the open ocean waters [32]; however, this is only a first tentative hypothesis as conditions, locations, and times of sampling vary greatly and vitamin concentrations in the ocean appear to be highly variable and dependent on many factors. The vitamin concentrations presented in this study are, to our knowledge, the first published values for a seawater sample from the North Sea. The values of B_3_, DMB, and Cbi are the first reported concentrations for seawater samples ever.

### Vitamin content in a bacterial cell extract

*P. inhibens* (DSM 17395), a model organism of the Roseobacter group, was cultivated without the addition of vitamins or vitamin precursors. Cells were harvested at the beginning of the exponential growth phase for the detection of intracellular vitamins or respective precursors (ESM Fig. S13). Three biological replicates and two technical replicates were extracted and analyzed with the three above mentioned measurement techniques SRM, PRM, and full scan. For these experiments, α-ribazole as a potential precursor of vitamin B_12_ was added to the portfolio of analytes. To the best of our knowledge, the results shown here are the first de novo synthesized intracellular concentrations per cell for this broad range of vitamins. Besides HB_12_ and Cbi, all analytes were found in very different amounts (Table 4 and ESM Table S6 for molecules per cell). Levels of B_7_, CB_12_, MB_12_, and HET were extremely low (< 0.1 zmole per cell), whereas B_1_, B_3_, B_5_, and DMB ranged from about 5 to 267 zmole per cell. In terms of the applicability of SRM, PRM, and full scan to the quantification of vitamins in the cell extract, all three methods provide very similar results. Only for B_1_, B_2_, B_3_, B_5_, B_7_, and DB_7_ we detected slight differences in the concentrations, but always in the same order of magnitude. The three compared methods can all be used for the quantification of vitamins in bacteria. All vitamins measured here were synthesized de novo by *P. inhibens* and can serve as a benchmark for how many vitamins prototrophic bacteria can synthesize at ideal laboratory conditions.

**Table 4:** Intracellular amount of 17 different B vitamins and precursors in zeptomole (10^−21^ mol) per cell *P. inhibens*

	Zeptomole (10^−21^ mol) per cell ± SD		
Vitamin	TSQ SRM	Orbitrap PRM	Orbitrap full scan
B_1_	35.53 ± 12.26	27.59 ± 7.68	31.05 ± 9.96
B_2_	3.04 ± 0.67	4.78 ± 2.15	4.23 ± 1.65
B_3_	267.27 ± 64.96	233.01 ± 47.46	209.36 ± 46.39
B_5_	4.96 ± 1.93	11.02 ± 4.77	4.72 ± 2.44
B_6_	1.15 ± 0.17	1.11 ± 0.16	1.12 ± 0.15
B_7_	0.08 ± 0.01	0.02 ± 0.01	0.02 ± 0.01
B_9_	0.65 ± 0.20	0.79 ± 0.30	0.74 ± 0.29
CB_12_	nd	0.02 ± 0.01	0.02 ± 0.01
AB_12_	0.91 ± 0.14	1.03 ± 0.13	0.95 ± 0.12
MB_12_	0.09 ± 0.04	0.09 ± 0.04	0.09 ± 0.04
HB_12_	nd	nd	nd
HMP	2.16 ± 1.17	2.07 ± 1.19	2.09 ± 1.13
HET	0.03 ± 0.01	0.02 ± 0.01	0.02 ± 0.01
DB_7_	1.33 ± 0.15	0.97 ± 0.13	1.07 ± 0.12
DMB	8.26 ± 0.92	8.01 ± 0.65	7.84 ± 0.76
Cbi	nd	nd	nd
α-Rib	0.32 ± 0.05	0.32 ± 0.05	0.31 ± 0.05

*nd*, not detected

*SD*, standard deviation

While the seawater sample shows a fairly balanced distribution, surprisingly, we detected very high concentrations of B_3_ and also B_1_ in the bacterial cell extract (ESM Fig. S14). The high B_3_ content in *P. inhibens* is comparable to that found in gut bacteria [33, 34]. Presumably, B_3_ is required at high quantities since it is a precursor of nicotinamide adenine dinucleotide (NAD), which is required, among other things, as an ubiquitous and important hydrogen carrier in numerous redox reactions [35]. B_1_ plays an important role in the formation and breaking of carbon–carbon-bonds, and relatively large amounts have been reported in other marine bacteria [12, 36]. Tang et al. [37] investigated the vitamin demand of ten auxotrophic microalgae. Required amounts for the growth of one cell were subject to strong fluctuations among the tested isolates (B_1_ (6.52*10^−21^–1.94*10^−17^ mol per cell), B_7_ (1.12*10^−21^–3.19*10^−19^ mol per cell), and B_12_ (1.79*10^−22^–1.66*10^−18^ mol per cell)). Intracellular vitamin concentrations of *P. inhibens* range at approx. 30*10^−21^ for B_1_ and approx. 1.1*10^−21^ for total B_12_ (Table 4). Thus, the intracellular B_1_ and B_12_ content of a single bacterial cell, in this case *P. inhibens*, can cover the vitamin demand of most of the tested eukaryotic unicellular organisms. In nature, de novo synthesized vitamins can be released in different ways. On the one hand, vitamins can be actively excreted by living organism and on the other hand, they can be set free by cell death through virus infection [38], cell lysis [39], or sloppy feeding [40]. Heal et al. [9] examined various B_12_ producing prokaryotes for their B_12_ concentration per cell and also found AB_12_ to be the most abundant of the B_12_ variants. The prototrophs studied by Heal et al. [9] had no detectable or 2–7 times higher intracellular concentrations for total B_12_, compared to *P. inhibens*. In particular, the investigated archaeal representatives showed significantly elevated concentrations of B_12_, with highest values found in *Nitrosopumilus*. Together, our results and those of Heal et al. [9] show that prokaryotic isolates seem to have a different B_12_ synthesis potential [41]. Synthesis potential of other B vitamins by other prokaryotes or eukaryotes is still largely unknown. We believe that the concentrations per cell we found for all B vitamins and some biosynthetic precursors in *P. inhibens* can serve as a proxy for future studies. To better understand the relationship between marine microorganisms and the marine vitamin cycle, the vitamin demand of consumers and the synthesis potential of producers should be investigated more intensively.

### Potential of QqQMS and HRMS

Studies comparing QqQMS and HRMS, addressing the analysis of drugs in sewage, are in accordance with our findings that neither method is clearly better, in terms of detection limits, but HRMS can be as good as QqQMS, if not better [27, 42]. Kaufmann et al. and Henry et al. also reported that these two types of mass spectrometers do not differ much in their sensitivity and selectivity [22, 24]. Nevertheless, HRMS, with its high flexibility through identification using exact masses at high resolution and retrospective evaluation of full scan measurements, offers great potential for the future of pharmacological and environmental nontargeted analysis [22, 24]. The results of the given study on B vitamins and selected precursors show that HRMS full scan measurements, even in particular for a broad range of polarities and diverse compounds, surpass common QqQMS/SRM in its capabilities for quantitative trace analysis. In terms of handling and analytical potential, a HRMS instrument such as the Orbitrap Fusion used in this paper or a less expensive Q Exactive is equivalent to a QqQMS.

## Conclusion

With recently introduced high-resolution mass spectrometers emerging, QqQMS may no longer be the sole leader in trace analysis with LC/MS. Based on the analytical limits presented, it can be concluded that both QqQMS with SRM, and HRMS with PRM and in particular full scan are capable of detecting similarly low levels of B vitamins. A major advantage of HRMS full scan measurement is the reduced developmental effort, as it is not necessary to optimize each analyte individually for fragment ions and collision energies, which is definitely required for SRM/PRM. The exact molecular masses can be used for high-resolution identification and trace quantification of targeted vitamins of interest, but the resulting chromatograms can also be studied for a broader range of other compounds and even be re-evaluated for other compounds later. Matrix-related time shifts, highly relevant for SRM or PRM modes, loose importance. The applicability of the three methods presented for the trace quantification of B vitamins and selected precursors has been demonstrated using a seawater sample from the North Sea and a cell extract of *P. inhibens*, a bacterial representative of the Roseobacter group, where nearly all studied vitamins and precursors were found in different amounts. The results prove that the HRMS full scan method can be used to detect the same natural vitamin concentrations as a SRM method. For purely routine measurements using the same matrix and the same limited number of analytes, a QqQMS in SRM measurement mode is very well suited, since no varying matrix effects should occur. However, our results indicate that mass spectrometers with high resolution in full scan mode can be used for trace analysis and might in the future replace triple quadrupole instruments.

## Supplementary Information

Below is the link to the electronic supplementary material.

Supplementary file1 (PDF 2.74 MB)

## Data Availability

The datasets generated and analyzed during the current study are available from the corresponding author on reasonable request.
