# Supplementary material for: Simultaneous quantification of all B vitamins and selected biosynthetic precursors in seawater and bacteria by means of different mass spectrometric approaches
Source: Anal Bioanal Chem. 2022 Oct 5;414(27):7839–54. doi: 10.1007/s00216-022-04317-8 (PMC9568461; doi:10.1007/s00216-022-04317-8)
Supplement: Supplementary file 1 — Supplementary file1 (PDF 2.74 MB) [file 216_2022_4317_MOESM1_ESM.pdf]

**Simultaneous quantification of all B vitamins and certain biosynthetic precursors in seawater and bacteria by means of different mass spectrometric approaches**

Stefan Bruns[1], Gerrit Wienhausen[1], Barbara Scholz-Böttcher[1], Heinz Wilkes[1]

[1] Institute for Chemistry and Biology of the Marine Environment (ICBM), Carl von Ossietzky University of Oldenburg, 26129 Oldenburg, Germany

Corresponding author: Heinz Wilkes (heinz.wilkes@uni-oldenburg.de)

Analytical and Bioanalytical Chemistry

**Table S1** Concentrations of the vitamins in a standard mixture of the dilution series

| Vitamin | Concentration (ng/l) |
|---|---|
| $B_1$ | 5000 |
| $B_2$ | 5000 |
| $B_3$ | 8000 |
| $B_5$ | 4000 |
| $B_6$ | 1000 |
| $B_7$ | 2000 |
| $B_9$ | 600000 |
| $CB_{12}$ | 125000 |
| $AB_{12}$ | 80000 |
| $MB_{12}$ | 20000 |
| $HB_{12}$ | 25000 |
| HMP | 2500 |
| HET | 1000 |
| $DB_7$ | 1000 |
| DMB | 5000 |
| Cbi | 100000 |

**Table S2** Source parameters for both mass spectrometers

| | TSQ | Orbitrap |
|---|---|---|
| Vaporizer Temperature (°C) | 400 | 350 |
| Transfer Tube Temperature (°C) | 340 | 300 |
| Sheath Gas Pressure (arbitrary units) | 60 | 50 |
| Aux Gas Pressure (arbitrary units) | 20 | 10 |

| Vitamin | TSQ Spray Voltage (V) (+) | Orbitrap Spray Voltage (V) (+) |
|---|---|---|
| $B_1$ | 3000 | 2000 |
| $B_2$ | 3000 | 2500 |
| $B_3$ | 3000 | 3000 |
| $B_5$ | 3000 | 3000 |
| $B_6$ | 3000 | 3000 |
| $B_7$ | 3000 | 3000 |
| $B_9$ | 3000 | 3500 |
| $CB_{12}$ | 2000 | 3500 |
| $AB_{12}$ | 2000 | 3500 |
| $MB_{12}$ | 2000 | 3500 |
| $HB_{12}$ | 2000 | 3500 |
| HMP | 3000 | 2000 |
| HET | 3000 | 2000 |
| $DB_7$ | 3000 | 3000 |
| DMB | 3000 | 2000 |
| Cbi | 3000 | 3500 |

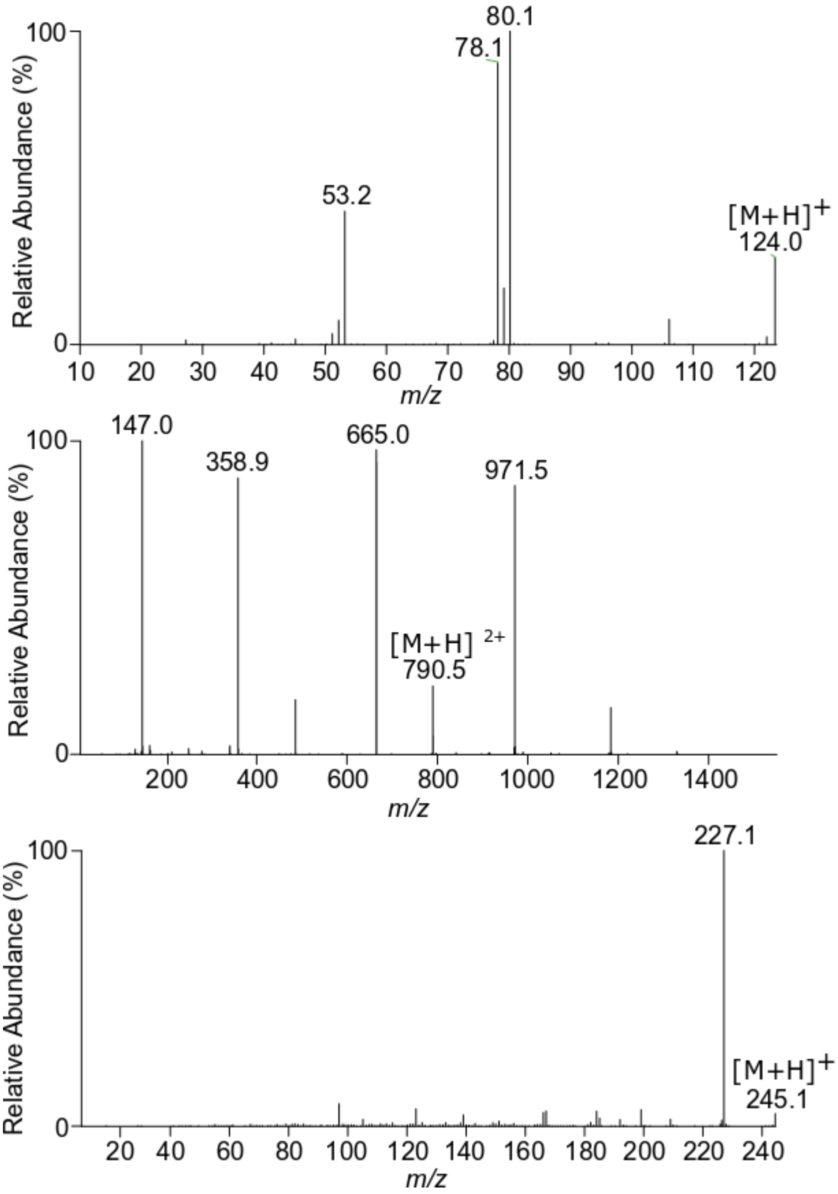


**Fig. S1** Fragmentation of $B_3$ with remaining molecular ion (top), fragmentation of $AB_{12}$ into multiple fragment ions of moderate intensity (middle), fragmentation of $B_7$ into one intense fragment ion (bottom). All spectra were measured on the TSQ Quantum

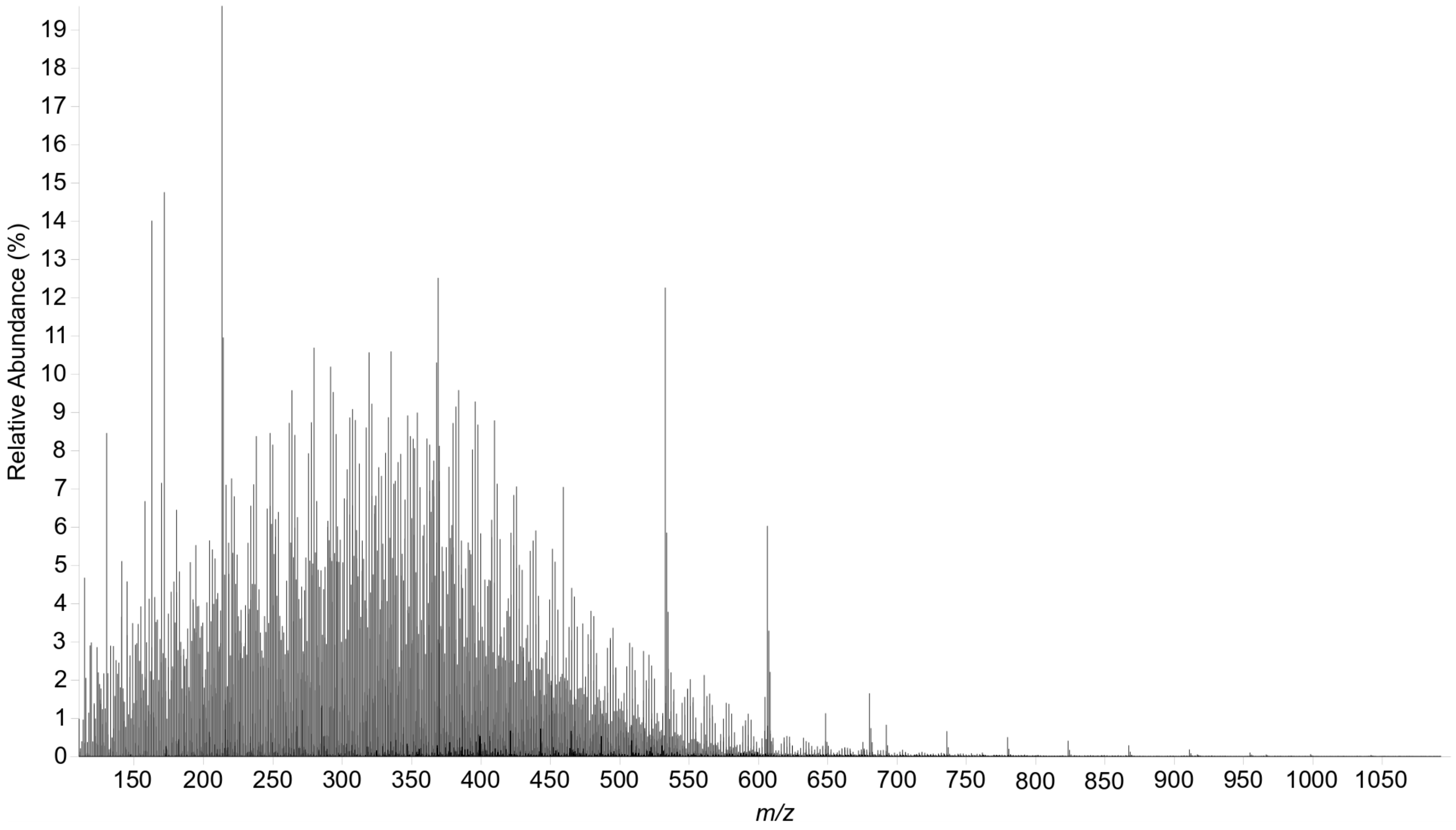


**Fig. S2** Summed mass spectrum of a full scan measurement of a seawater extract. The signal of a background contamination at *m/z* 214 was cut off to show the distribution of the remaining masses in the elution range of the vitamins

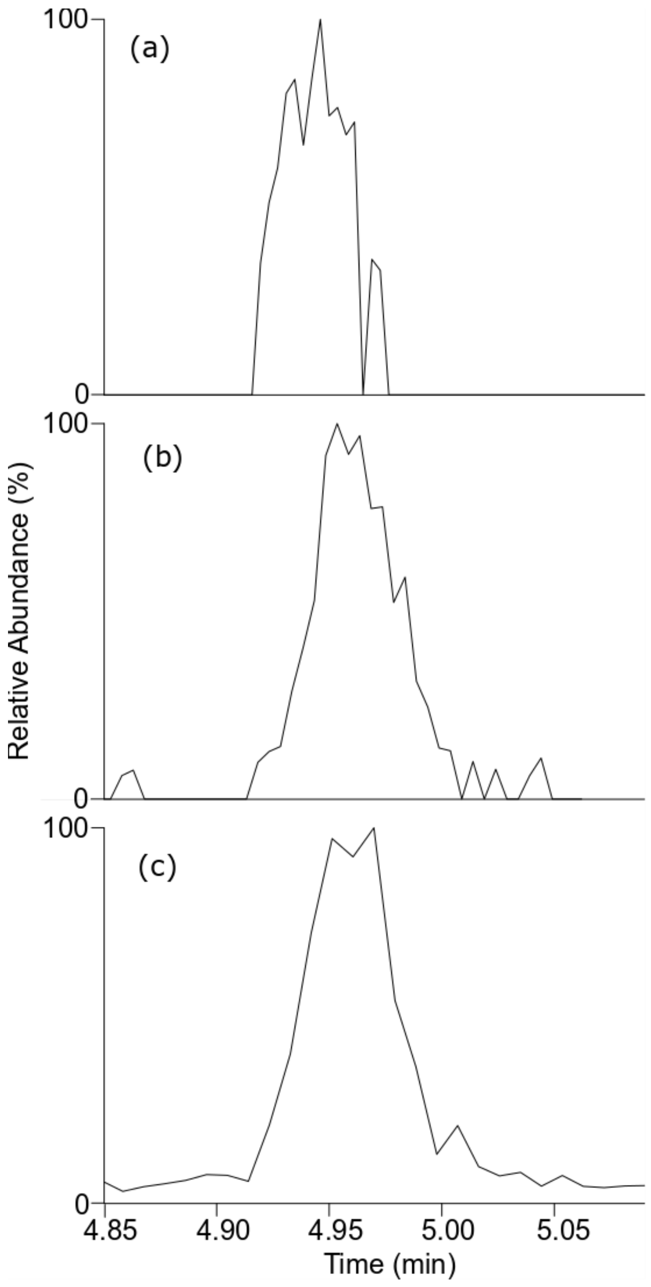

**Fig. S3** Signals of HET from PRM measurements with resolutions of (a) 15.000, (b) 60.000, and (c) 120.000

**Table S3** Instrumental limits of detection in ng/l and pg on column with 5 µl injection volume. Data represent the lowest concentration that gave a reproducible signal in the linear range

| | LOD TSQ SRM | | LOD Orbitrap PRM | | LOD Orbitrap full scan | |
|---|---|---|---|---|---|---|
| Vitamin | ng/l | pg on column | ng/l | pg on column | ng/l | pg on column |
| $B_1$ | 1000 | 5.00 | 20000 | 100.00 | 20000 | 100.00 |
| $B_2$ | 50 | 0.25 | 50 | 0.25 | 50 | 0.25 |
| $B_3$ | 500 | 2.50 | 100 | 0.50 | 50 | 0.25 |
| $B_5$ | 10 | 0.05 | 50 | 0.25 | 100 | 0.50 |
| $B_6$ | 20 | 0.10 | 20 | 0.10 | 50 | 0.25 |
| $B_7$ | 50 | 0.25 | 50 | 0.25 | 200 | 1.00 |
| $B_9$ | 500 | 2.50 | 200 | 1.00 | 200 | 1.00 |
| $CB_{12}$ | 500 | 2.50 | 100 | 0.50 | 100 | 0.50 |
| $AB_{12}$ | 500 | 2.50 | 100 | 0.50 | 100 | 0.50 |
| $MB_{12}$ | 200 | 1.00 | 200 | 1.00 | 50 | 0.25 |
| $HB_{12}$ | 20000 | 100.00 | 20000 | 100.00 | 50000 | 250.00 |
| HMP | 20 | 0.10 | 100 | 0.50 | 50 | 0.25 |
| HET | 10 | 0.05 | 10 | 0.05 | 10 | 0.05 |
| $DB_7$ | 10 | 0.05 | 20 | 0.10 | 20 | 0.10 |
| DMB | 10 | 0.05 | 20 | 0.10 | 50 | 0.25 |
| Cbi | 500 | 2.50 | 1000 | 5.00 | 1000 | 5.00 |

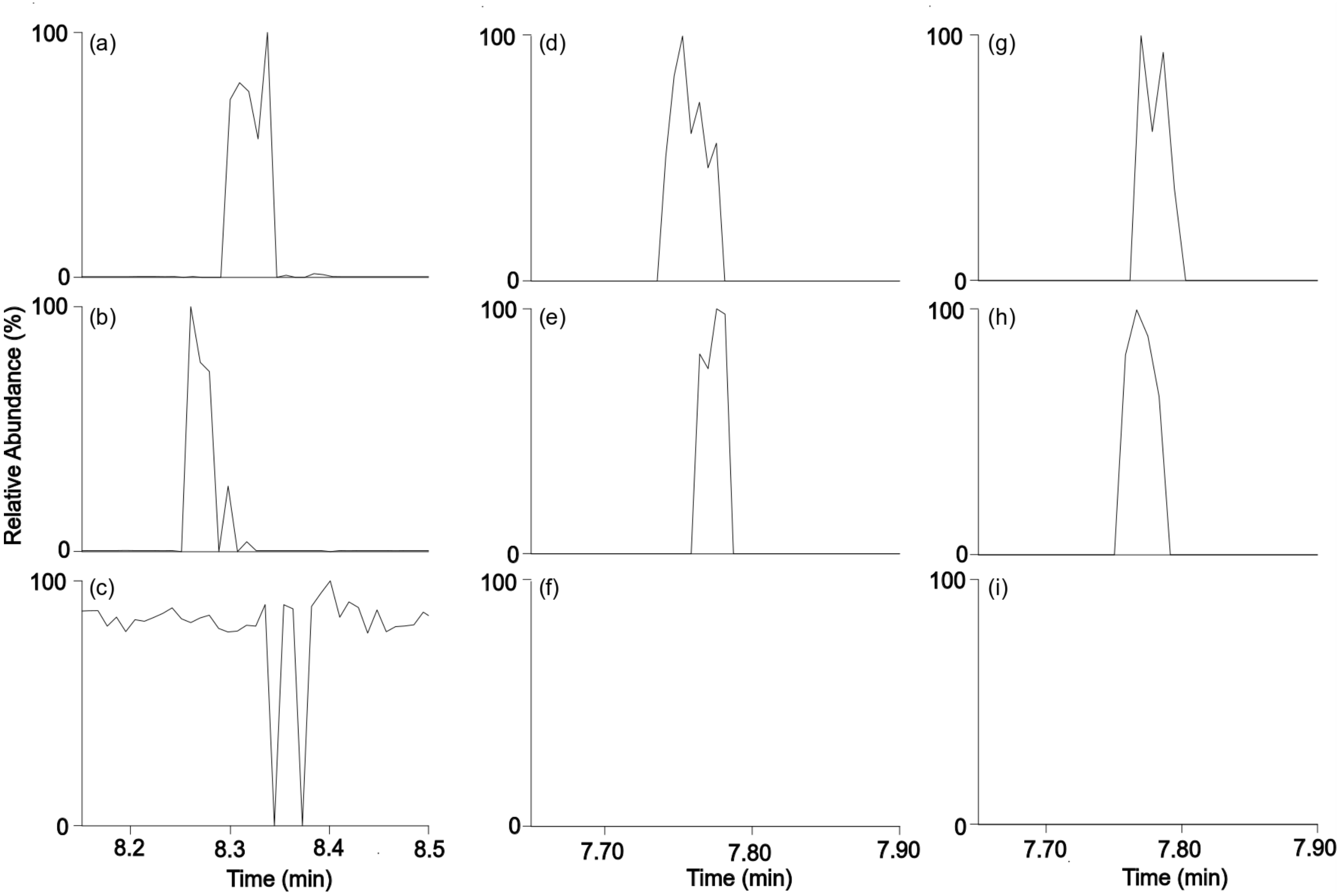


**Fig. S4** EIC of $CB_{12}$ with concentrations of (a) 250 pg/ml, (b) 125 pg/ml, (c) 25 pg/ml with SRM at the TSQ; (d) 100 pg/ml, (e) 50 pg/ml, (f) 20 pg/ml with PRM at the Orbitrap and (g) 50 pg/ml, (h) 20 pg/ml, (i) 10 pg/ml with full scan at the Orbitrap. For the HRMS measurements, no noise is seen besides the analyte peak

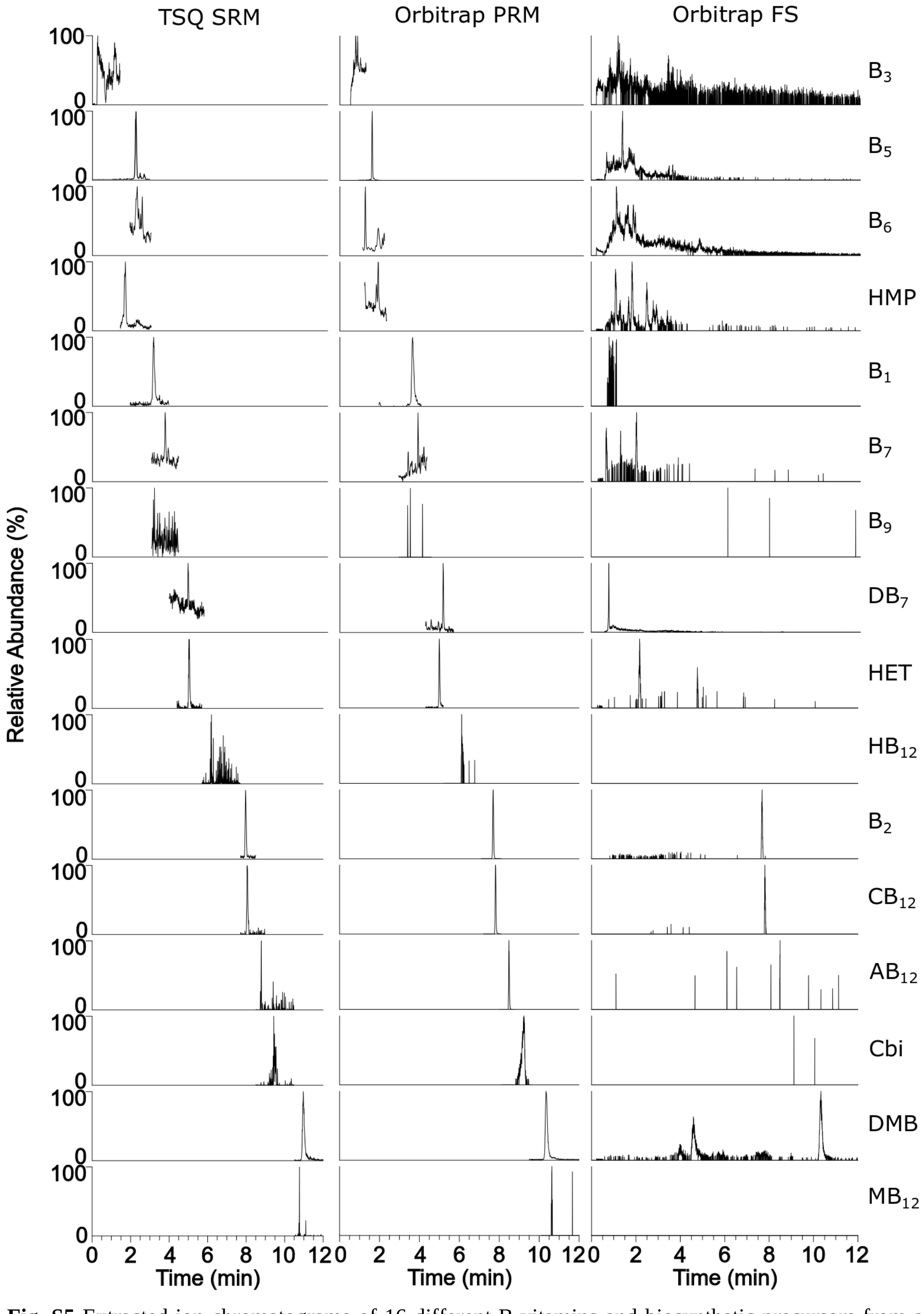


**Fig. S5** Extracted ion chromatograms of 16 different B vitamins and biosynthetic precursors from a North Sea water sample, concentrated by solid phase extraction, measured with three different MS methods (selected reaction monitoring (SRM), parallel reaction monitoring (PRM) and full scan (FS))

**Table S4** Retention times of individual vitamins at TSQ and Orbitrap in the presence or absence of matrix

| | TSQ | | Orbitrap | |
|---|---|---|---|---|
| Vitamin | no Matrix | with Matrix | no Matrix | with Matrix |
| $B_3$ | 1.11 | 1.11 | 0.83 | 0.85 |
| $B_5$ | 1.60 | 1.90 | 1.24 | 1.55 |
| $B_6$ | 2.26 | 2.29 | 1.76 | 1.84 |
| HMP | 2.33 | 1.66 | 1.68 | 1.06 |
| $B_1$ | 2.89 | 3.25 | 2.90 | 3.51 |
| $B_7$ | 3.58 | 3.99 | 3.95 | 4.13 |
| $B_9$ | 3.61 | 3.84 | 4.02 | 3.95 |
| $DB_7$ | 4.78 | 5.01 | 4.91 | 5.02 |
| HET | 5.13 | 5.22 | 4.99 | 4.98 |
| $B_2$ | 8.08 | 8.18 | 7.69 | 7.69 |
| $CB_{12}$ | 8.18 | 8.25 | 7.80 | 7.81 |
| $AB_{12}$ | 8.90 | 9.02 | 8.47 | 8.49 |
| Cbi | 9.63 | 9.78 | 9.22 | 9.24 |
| DMB | 11.00 | 11.13 | 10.27 | 10.36 |
| $MB_{12}$ | 11.03 | 11.09 | 10.61 | 10.62 |
| $HB_{12}$ | 6.59 | 6.91 | 6.00 | 6.12 |

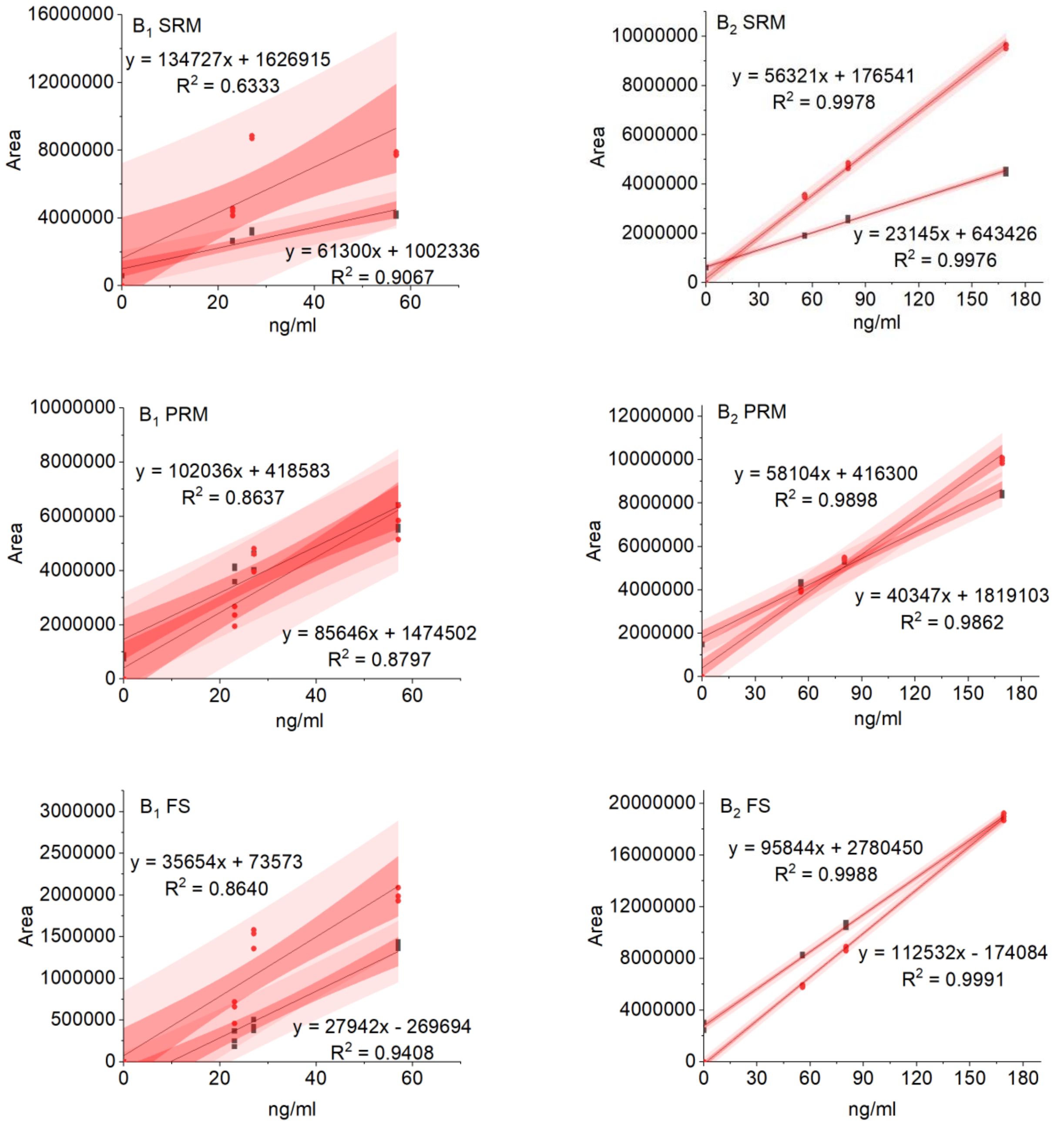


**Fig. S6** Regression lines illustrating linear response and calibration behavior of the respective analytes. Furthermore, ion suppression via addition of known amounts of $B_1$ (left) and $B_2$ (right) to ultrapure water (red circles) and a processed seawater sample (black squares) is reflected by the decline in slope. The three methodological approaches compared are: SRM on the TSQ Quantum, PRM and FS on the Orbitrap Fusion, respectively. The regression lines in black with the linear equations and the squared correlation coefficients are displayed as well as the 95% confidence (dark red) and prediction (light red) bands

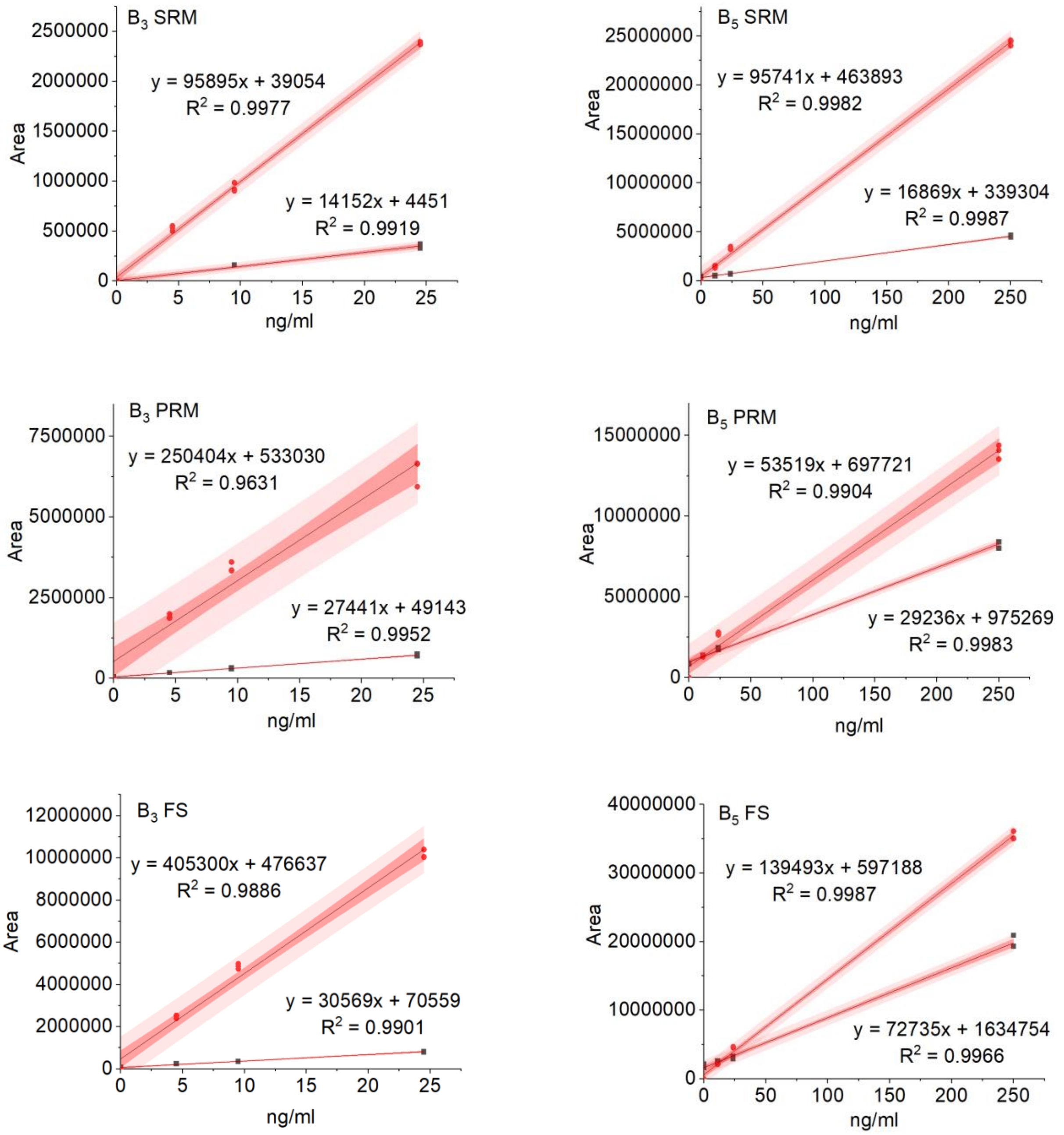


**Fig. S7** Regression lines illustrating linear response and calibration behavior of the respective analytes. Furthermore, ion suppression via addition of known amounts of $B_3$ (left) and $B_5$ (right) to ultrapure water (red circles) and a processed seawater sample (black squares) is reflected by the decline in slope. The three methodological approaches compared are: SRM on the TSQ Quantum, PRM and FS on the Orbitrap Fusion, respectively. The regression lines in black with the linear equations and the squared correlation coefficients are displayed as well as the 95% confidence (dark red) and prediction (light red) bands

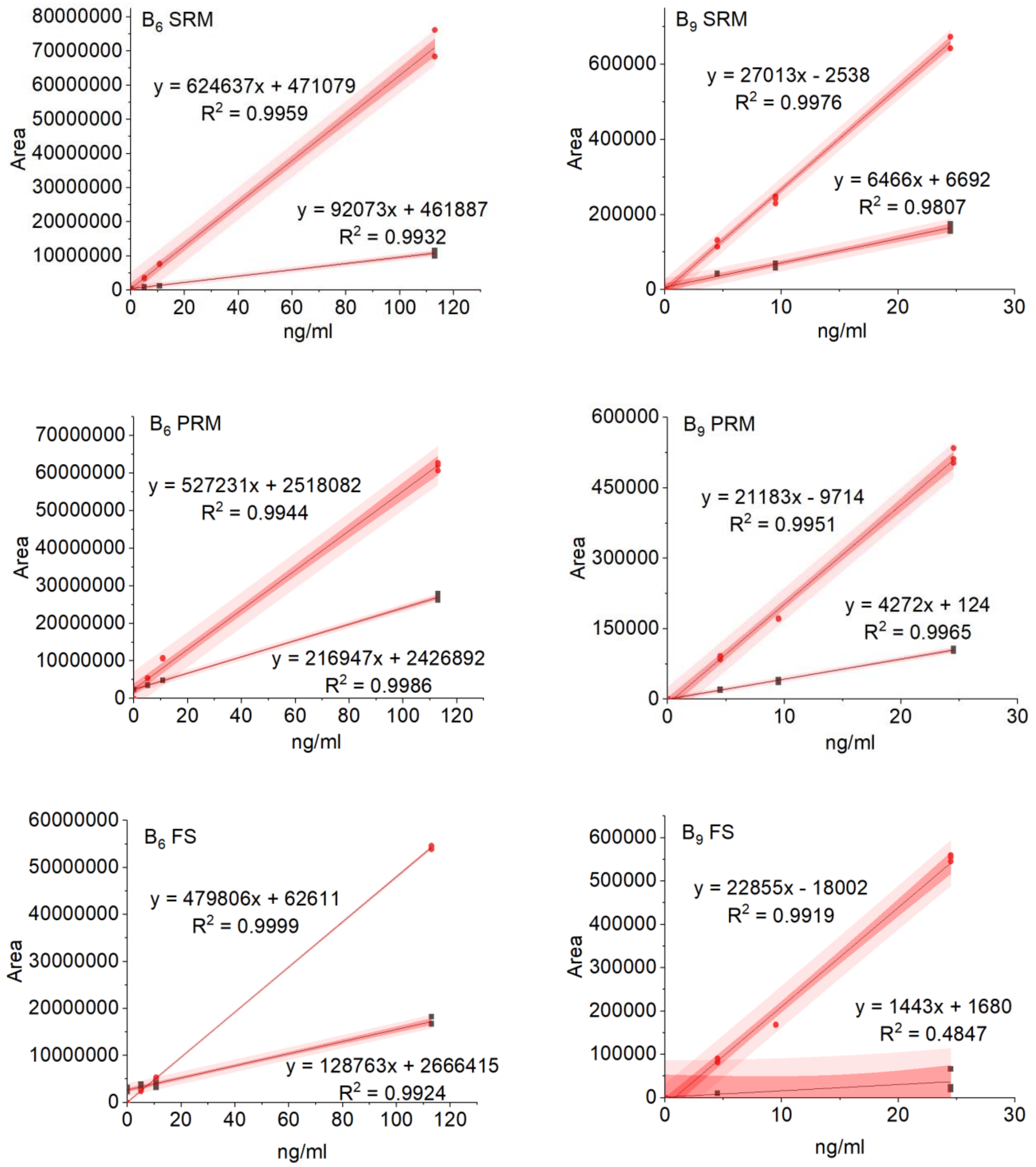


**Fig. S8** Regression lines illustrating linear response and calibration behavior of the respective analytes. Furthermore, ion suppression via addition of known amounts of $B_6$ (left) and $B_9$ (right) to ultrapure water (red circles) and a processed seawater sample (black squares) is reflected by the decline in slope. The three methodological approaches compared are: SRM on the TSQ Quantum, PRM and FS on the Orbitrap Fusion, respectively. The regression lines in black with the linear equations and the squared correlation coefficients are displayed as well as the 95% confidence (dark red) and prediction (light red) bands

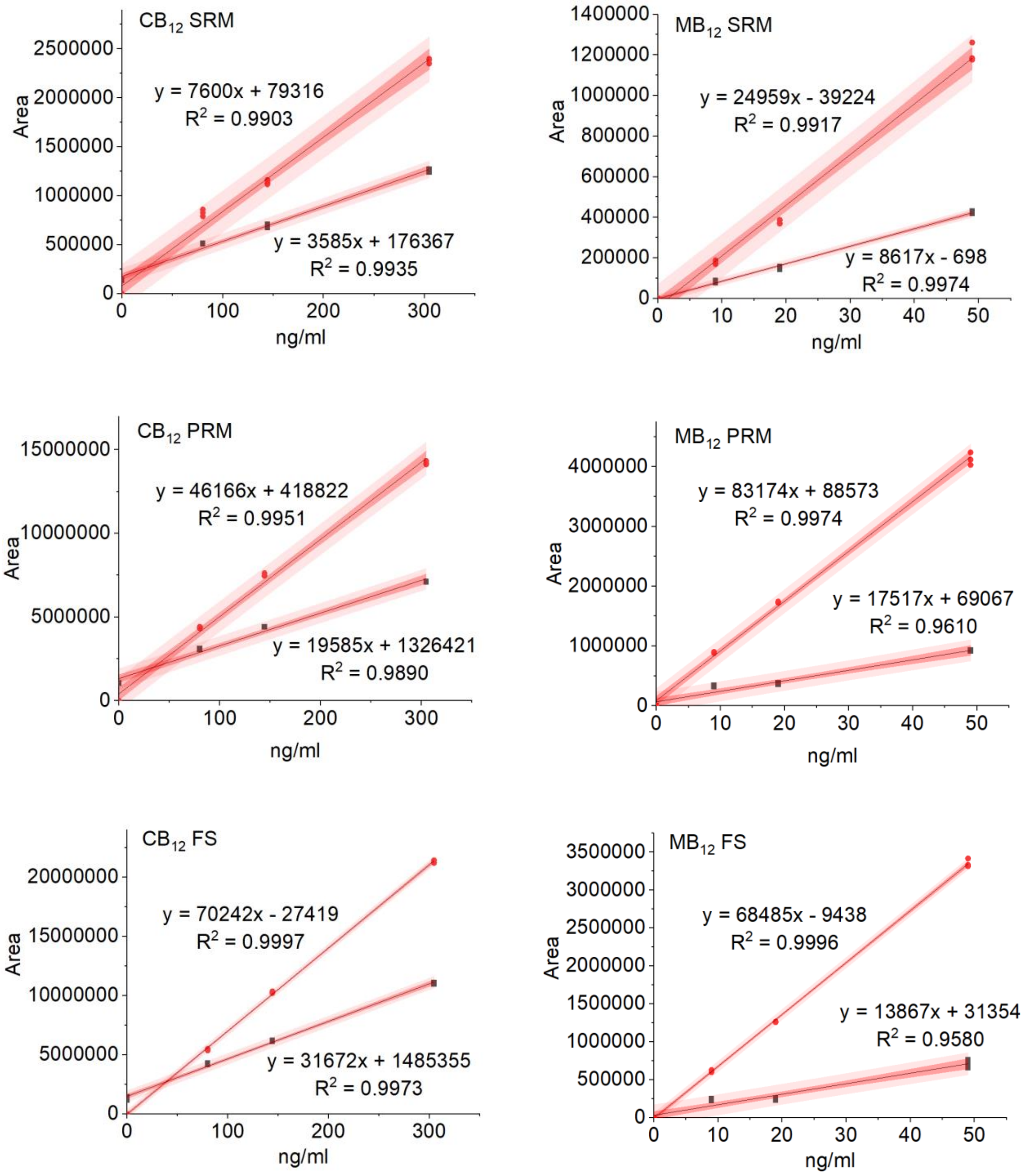


**Fig. S9** Regression lines illustrating linear response and calibration behavior of the respective analytes. Furthermore, ion suppression via addition of known amounts of $CB_{12}$ (left) and $MB_{12}$ (right) to ultrapure water (red circles) and a processed seawater sample (black squares) is reflected by the decline in slope. The three methodological approaches compared are: SRM on the TSQ Quantum, PRM and FS on the Orbitrap Fusion, respectively. The regression lines in black with the linear equations and the squared correlation coefficients are displayed as well as the 95% confidence (dark red) and prediction (light red) bands

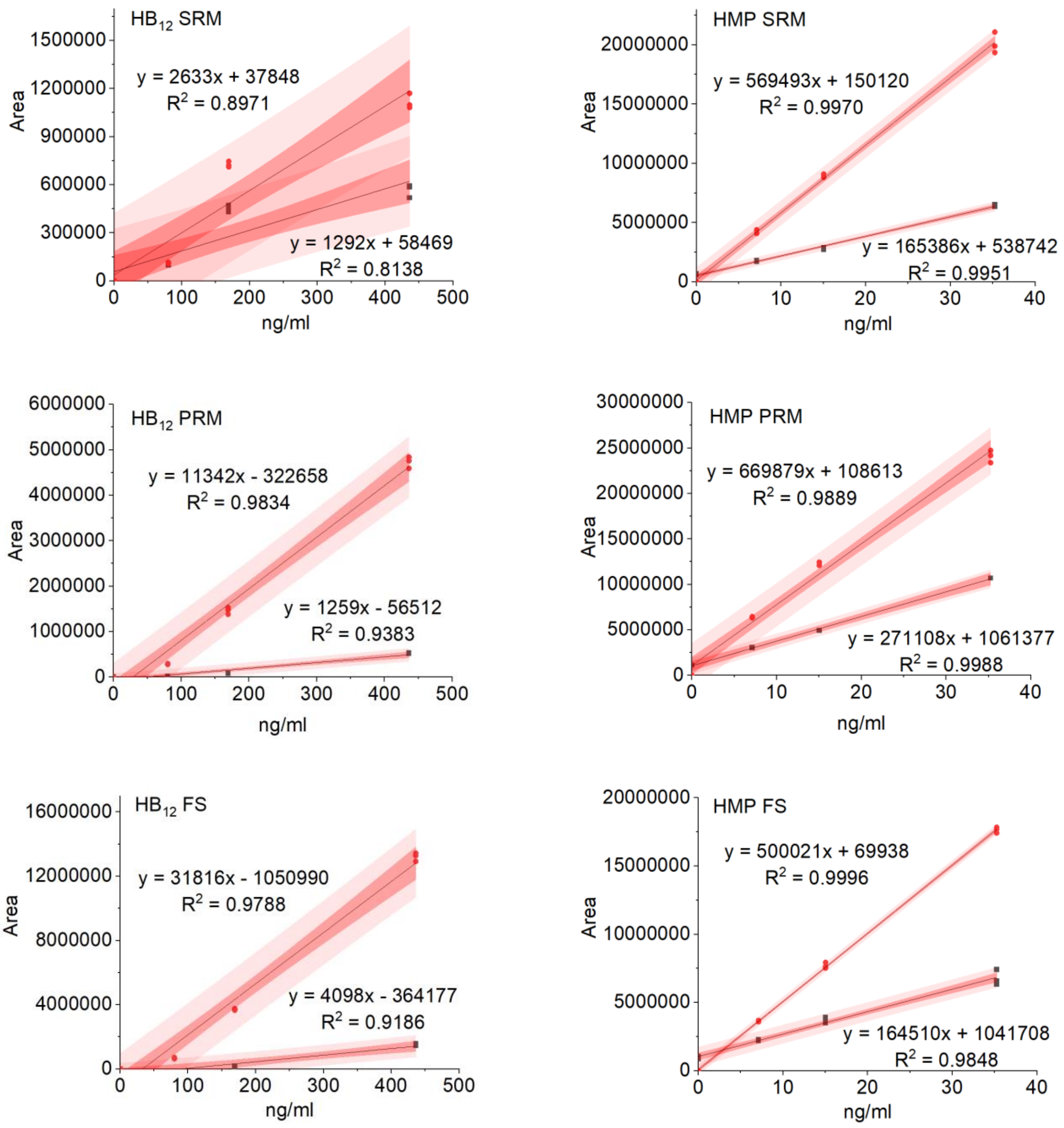


**Fig. S10** Regression lines illustrating linear response and calibration behavior of the respective analytes. Furthermore, ion suppression via addition of known amounts of $HB_{12}$ (left) and HMP (right) to ultrapure water (red circles) and a processed seawater sample (black squares) is reflected by the decline in slope. The three methodological approaches compared are: SRM on the TSQ Quantum, PRM and FS on the Orbitrap Fusion, respectively. The regression lines in black with the linear equations and the squared correlation coefficients are displayed as well as the 95% confidence (dark red) and prediction (light red) bands

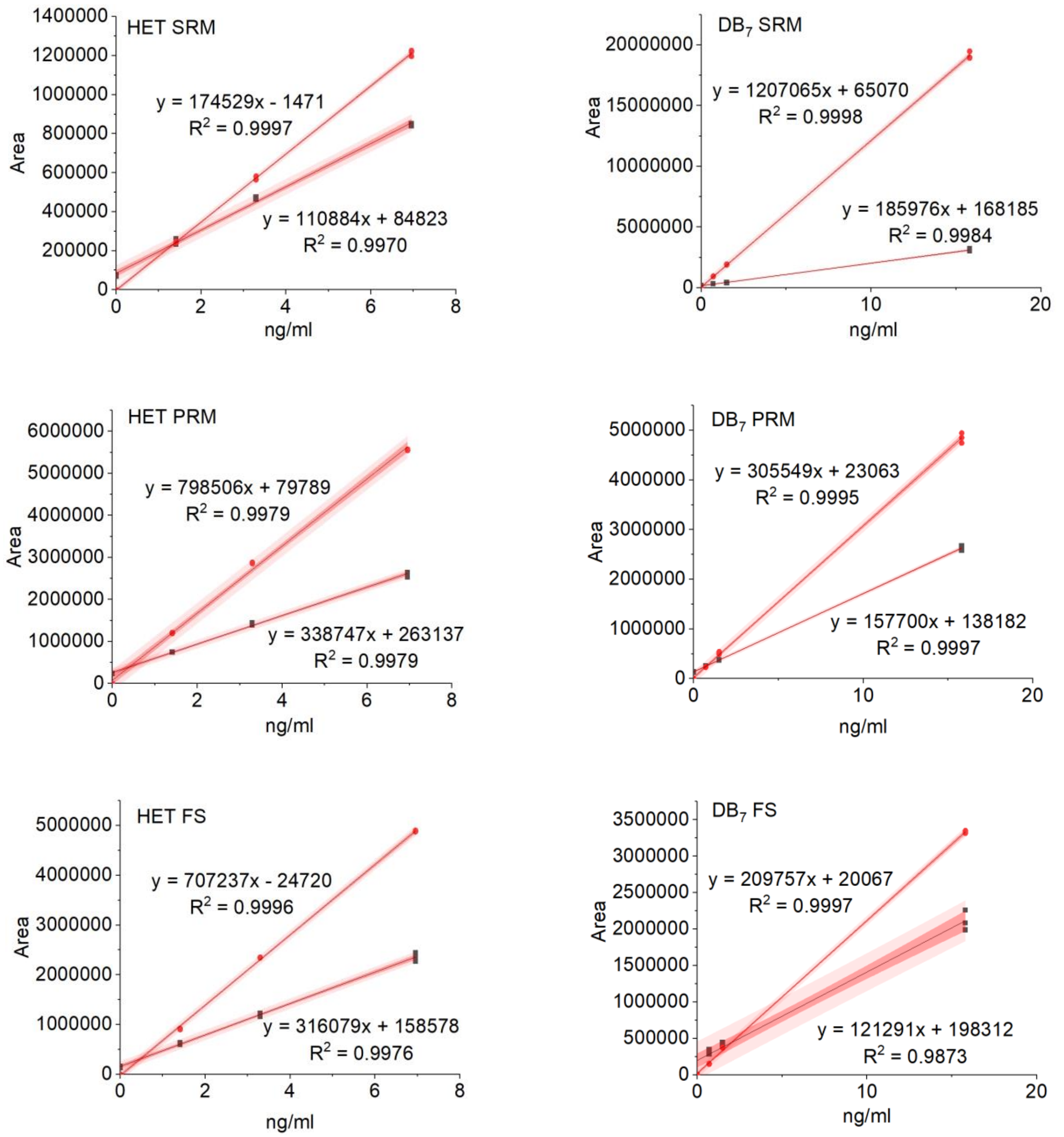


**Fig. S11** Regression lines illustrating linear response and calibration behavior of the respective analytes. Furthermore, ion suppression via addition of known amounts of HET (left) and $DB_7$ (right) to ultrapure water (red circles) and a processed seawater sample (black squares) is reflected by the decline in slope. The three methodological approaches compared are: SRM on the TSQ Quantum, PRM and FS on the Orbitrap Fusion, respectively. The regression lines in black with the linear equations and the squared correlation coefficients are displayed as well as the 95% confidence (dark red) and prediction (light red) bands

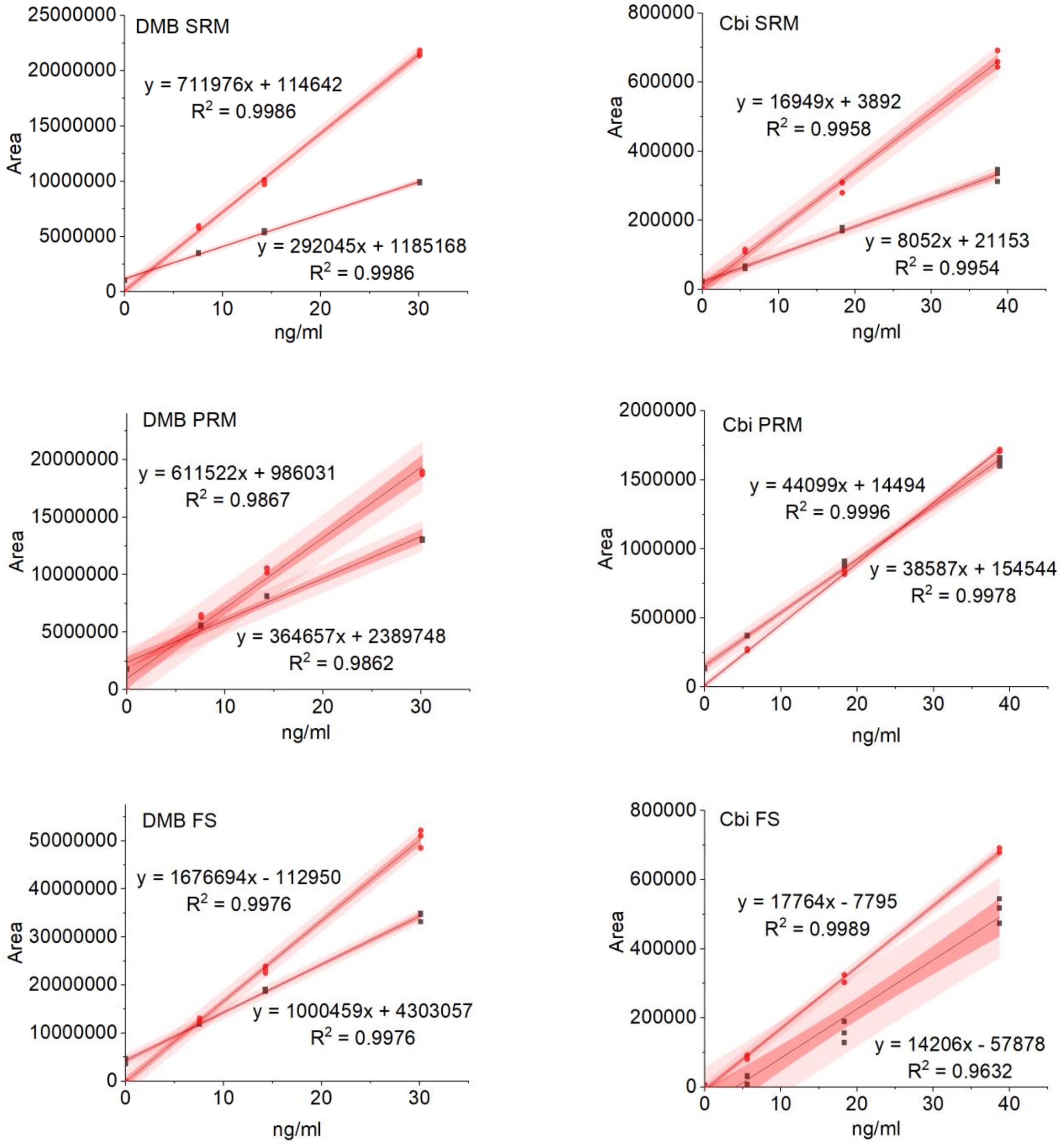


**Fig. S12** Regression lines illustrating linear response and calibration behavior of the respective analytes. Furthermore, ion suppression via addition of known amounts of DMB (left) and Cbi (right) to ultrapure water (red circles) and a processed seawater sample (black squares) is reflected by the decline in slope. The three methodological approaches compared are: SRM on the TSQ Quantum, PRM and FS on the Orbitrap Fusion, respectively. The regression lines in black with the linear equations and the squared correlation coefficients are displayed as well as the 95% confidence (dark red) and prediction (light red) bands

**Table S5** Vitamin content in a seawater sample from the North Sea in pM

| Vitamin | SRM (pM) | PRM (pM) | full scan (pM) |
|---|---|---|---|
| $B_1$ | 26.8 ± 7.5 | 16.6 ± 5.3 | nd |
| $B_2$ | 35.9 ± 2.4 | 31.9 ± 3.5 | 18.6 ± 0.9 |
| $B_3$ | nd | 26.0 ± 4.3 | 46.3 ± 9.6 |
| $B_5$ | 97.2 ± 8.2 | 53.4 ± 3.1 | 38.8 ± 4.5 |
| $B_6$ | 40.2 ± 12.4 | 31.3 ± 2.1 | 91.0 ± 8.6 |
| $B_7$ | 29.9 ± 2.5 | 0.8 ± 0.6 | nd |
| $B_9$ | nd | nd | nd |
| $CB_{12}$ | 15.4 ± 1.7 | 22.1 ± 2.5 | 15.4 ± 1.2 |
| $AB_{12}$ | 0.6 ± 0.1 | 1.1 ± 0.2 | 0.4 ± 0.2 |
| $MB_{12}$ | nd | 2.3 ± 1.3 | nd |
| $HB_{12}$ | nd | nd | nd |
| HMP | 15.8 ± 2.4 | 12.9 ± 1.8 | 27.3 ± 4.2 |
| HET | 1.4 ± 0.2 | 2.8 ± 0.2 | 1.4 ± 0.2 |
| $DB_7$ | 5.6 ± 0.7 | 1.4 ± 0.1 | nd |
| DMB | 13.7 ± 0.8 | 13.7 ± 1.7 | 9.6 ± 0.8 |
| Cbi | 1.1 ± 0.2 | 0.9 ± 0.1 | 0.7 ± 0.4 |

nd = not detected

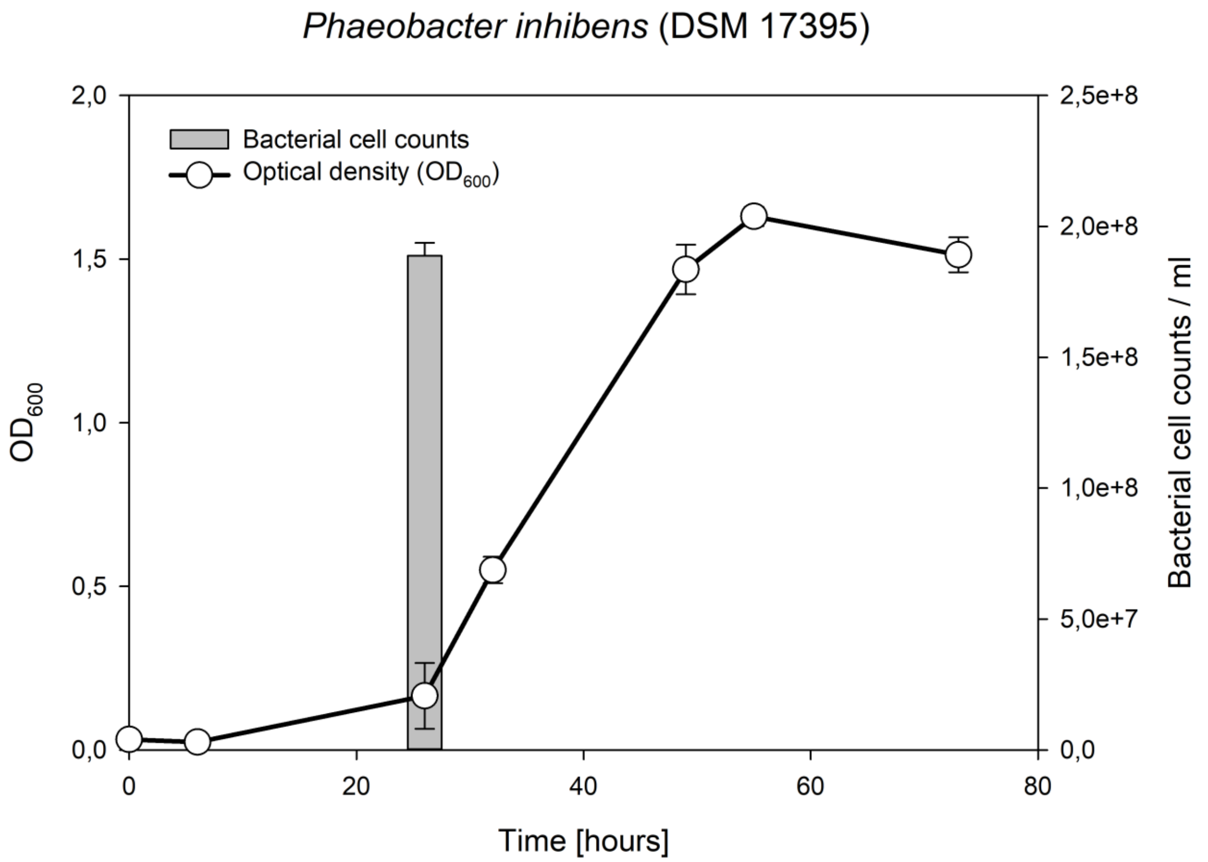


**Fig. S13** Growth curve of *P. inhibens* (DSM 17395), determined by optical density (circles). The cell counts per ml at the time of sampling are shown

**Table S6** Intracellular amount of 17 different B vitamins and biosynthetic precursors in molecules per cell *P. inhibens*

| | molecules per cell ± SD | | |
|---|---|---|---|
| Vitamin | TSQ SRM | Orbitrap PRM | Orbitrap full scan |
| $B_1$ | 21393 ± 6394 | 16616 ± 3962 | 18701 ± 5077 |
| $B_2$ | 1829 ± 349 | 2879 ± 954 | 2550 ± 763 |
| $B_3$ | 160950 ± 30245 | 140318 ± 22659 | 126079 ± 23554 |
| $B_5$ | 2988 ± 988 | 6635 ± 2387 | 2841 ± 1069 |
| $B_6$ | 690 ± 74 | 668 ± 75 | 673 ± 67 |
| $B_7$ | 49 ± 4 | 14 ± 2 | 14 ± 2 |
| $B_9$ | 391 ± 106 | 475 ± 158 | 448 ± 148 |
| $CB_{12}$ | nd | 10 ± 2 | 9 ± 2 |
| $AB_{12}$ | 549 ± 79 | 623 ± 58 | 574 ± 55 |
| $MB_{12}$ | 55 ± 21 | 57 ± 24 | 55 ± 22 |
| $HB_{12}$ | nd | nd | nd |
| HMP | 1299 ± 616 | 1245 ± 619 | 1260 ± 589 |
| HET | 17 ± 6 | 15 ± 6 | 13 ± 5 |
| $DB_7$ | 802 ± 90 | 584 ± 66 | 645 ± 69 |
| DMB | 4976 ± 523 | 4824 ± 367 | 4723 ± 421 |
| Cbi | nd | nd | nd |
| αRib | 194 ± 26 | 192 ± 27 | 185 ± 25 |

nd = not detected

SD = standard deviation

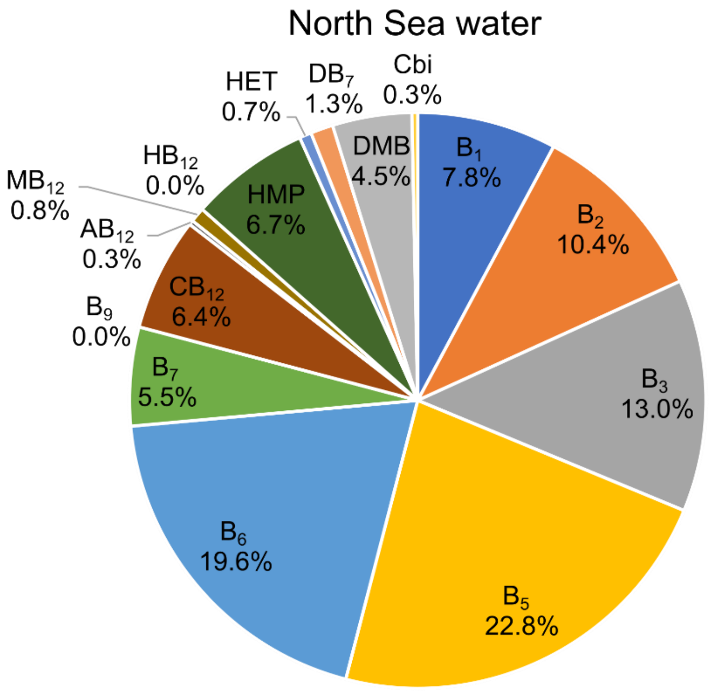


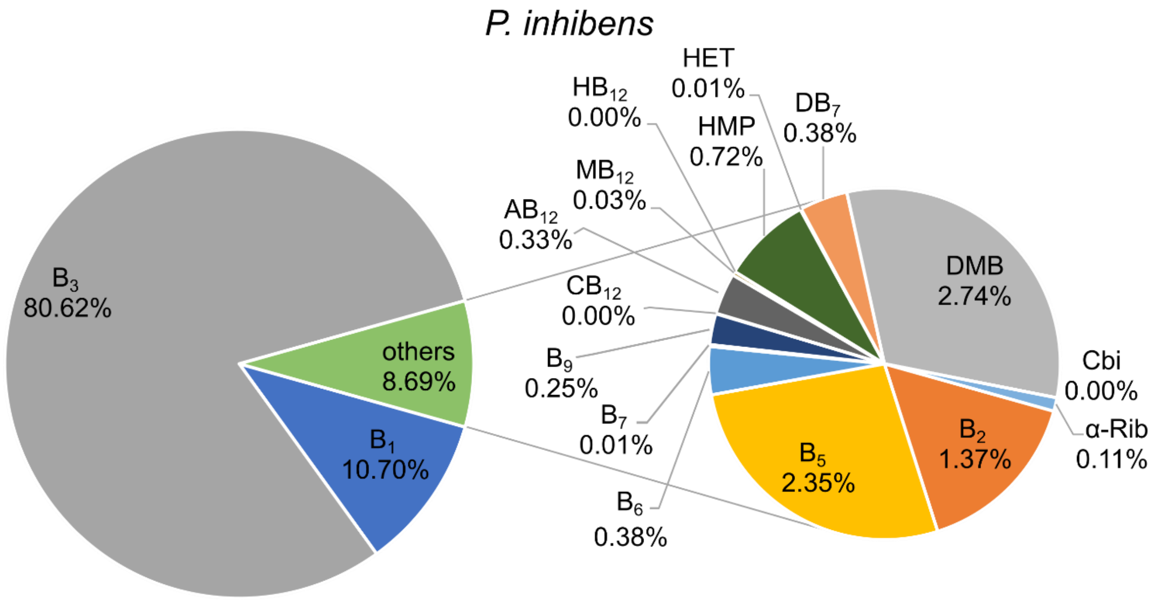


**Fig. S14** Percentage of all vitamins and precursors found in a seawater sample from the North Sea (top) and in an extract of cells of the bacterium *P. inhibens* (bottom). Display of all analytes found separately without $B_1$ and $B_3$ (bottom right)
